# Inherited chromosomally integrated human herpesvirus 6: regional variation in prevalence, association with angina, and identification of ancestral viral lineages in two large UK studies

**DOI:** 10.1128/jvi.02160-24

**Published:** 2025-06-05

**Authors:** Michael L. Wood, Adam J. Bell, Robin Young, Christopher Brownlie, Nick Orr, Archie Campbell, Jenna Nichols, Konstantinos Papageorgiou, Annette Lake, Nicolas M. Suarez, Katherine Smollett, Natasha Jesudason, Salvatore Camiolo, Sreenu Vattipally, Joseph Hughes, Kirby Brown, Leah M. Hunter, Euan Shaw, Skye Storrie, Rithu Paul Stansilaus, Eillis Sweeney, Tingyi Zhu, Angie Fawkes, Lee Murphy, William Tyne, Philip Howard, Michael E. Jones, Katarzyna Tomczyk, Anne Richmond, James F. Wilson, Duncan A. Clark, Christian Delles, Nicola Royle, Shona M. Kerr, Ana da Silva Filipe, Andrew J. Davison, Alex McConnachie, Anthony J. Swerdlow, Caroline Hayward, Ruth F. Jarrett

**Affiliations:** 1 MRC-University of Glasgow Center for Virus Research 155698, Glasgow, United Kingdom; 2 Robertson Center for Biostatistics, School of Health and Wellbeing, University of Glasgow 3526, Glasgow, United Kingdom; 3 The Institute of Cancer Research 5053, London, United Kingdom; 4 MRC Human Genetics Unit, IGC, University of Edinburgh 3124, Edinburgh, United Kingdom; 5 Center for Genomic and Experimental Medicine, IGC, University of Edinburgh 3124, Edinburgh, United Kingdom; 6 Usher Institute, University of Edinburgh 172239, Edinburgh, United Kingdom; 7 Edinburgh Clinical Research Facility, University of Edinburgh 3124, Edinburgh, United Kingdom; 8 School of Biological and Chemical Sciences, Queen Mary University of London 4617, London, United Kingdom; 9 School of Infection and Immunity, University of Glasgow 3526, Glasgow, United Kingdom; 10 School of Cardiovascular and Metabolic Health, University of Glasgow 3526, Glasgow, United Kingdom; 11 University of Leicester 4488, Leicester, United Kingdom; Cornell University Baker Institute for Animal Health, Ithaca, New York, USA

**Keywords:** herpesvirus, human herpesvirus 6, HHV-6, inherited HHV-6, iciHHV-6, telomere, chromosomal integration, generation Scotland, angina

## Abstract

Tens of millions of people worldwide have inherited chromosomally integrated human herpesvirus 6 (iciHHV-6), yet we know little about the consequences. iciHHV-6-positive individuals inherit the genome of HHV-6A or HHV-6B in the germline, and viral genomes are therefore present in every nucleated cell. To investigate the epidemiology of iciHHV-6 in the UK, almost 32,000 individuals were screened from two volunteer research studies: the family-based Generation Scotland: Scottish Family Health Study (GS:SFHS) and the Breakthrough Generations Study (BGS). iciHHV-6 prevalence in GS:SFHS was, to our knowledge, higher than that in other large studies at 2.74% (647/23,637), with an iciHHV-6B prevalence of 2.55%. Scottish participants were more likely to be iciHHV-6B-positive than English (*P* < 0.001), and the BGS results suggested a north-south gradient of iciHHV-6B prevalence in mainland Britain. Disease association analysis confirmed the previously reported association with angina, with an odds ratio of 1.91 (95% confidence interval, 1.29, 2.82) following adjustment for known risk factors, providing compelling evidence that iciHHV-6 contributes to the risk of a common symptom. *De novo* integrations were not detected within GS:SFHS pedigrees; rather, our findings indicated that three viral lineages accounted for over 95% of iciHHV-6A-positive samples, and six viral lineages accounted for 90% of iciHHV-6B-positive samples in GS:SFHS. This study demonstrates that iciHHV-6 is common in the UK, shows significant regional heterogeneity in prevalence, is not entirely harmless, and is largely derived from a relatively small number of ancestral viral lineages.

**IMPORTANCE:**

Human herpesvirus 6 (HHV-6) has the unusual ability to integrate into the host chromosome telomeres. Most of the world’s population is infected by HHV-6 in early childhood, but around 1% inherit the virus as a chromosomally integrated viral genome—referred to as inherited chromosomally integrated HHV-6 (iciHHV-6). Little is known about the consequences of iciHHV-6, which has the potential to cause disease through various mechanisms. Here, we have used large cohorts to study iciHHV-6 prevalence, lineages, and phenotypic associations. We replicate a previously reported association between iciHHV-6 and angina, suggesting that iciHHV-6 is not entirely benign. We show significant variation in iciHHV-6 prevalence within the UK with almost 3% of Scottish people carrying iciHHV-6. In the first detailed analysis of viral lineages at the population level, we show that 90% of iciHHV-6 is explained by nine ancestral viral lineages. These results have important implications for future disease association analyses.

## INTRODUCTION

Human herpesvirus 6A (HHV-6A) and human herpesvirus 6B (HHV-6B) are two closely related betaherpesviruses that can integrate into host chromosomal DNA, specifically into telomeres (1–3). Although this feature is shared with some other orthoherpesviruses (4), HHV-6A and HHV-6B are unique in their ability to stably integrate into germline DNA, giving rise to inherited chromosomally integrated HHV-6 (iciHHV-6) (5). Millions of people worldwide have iciHHV-6 (6), but little is known about this understudied phenomenon. Most iciHHV-6 genomes are intact and have the potential to reactivate *in vivo* (7–9). Therefore, iciHHV-6 is not simply a fossilized viral remnant, but a dynamic structure that can give rise to infectious virus and potentially interfere with telomere function (7–11).

Most individuals are infected by HHV-6A or HHV-6B, or both, as exogenous agents in childhood (12, 13). Primary infection by HHV-6B can cause roseola infantum and is a common cause of febrile seizures (14–16). Following primary infection, the virus persists for life and generally causes few problems in immunocompetent individuals (17). In contrast, viral reactivation in immunocompromised hosts can have serious consequences, including encephalitis, lower respiratory tract disease, and acute graft vs host disease (1, 17–20). Associations with other diseases, including multiple sclerosis, have been reported but not confirmed (21–24).

Although infection with exogenous HHV-6A or HHV-6B is almost ubiquitous, a smaller but substantial proportion of individuals have iciHHV-6. These individuals have one, or occasionally more, copies of the HHV-6A or HHV-6B genome integrated into every nucleated cell of their body, and the virus is transmitted to offspring in the germline (1, 25, 26). HHV-6 genomes consist of a long unique (U) region flanked by two almost identical direct repeats (DRs). The integration mechanism is not fully understood but appears to involve homology-mediated recombination between host telomeres, which comprise TTAGGG hexameric repeats, and a telomere repeat array in the right-hand DR (DR_R_). *In vivo* and *in vitro* studies provide evidence that some integrated viral genomes can be released from host chromosomes with full viral reactivation (2, 7–11, 27), and this is perhaps the most likely way in which iciHHV-6 could cause or contribute to disease. However, the presence or sudden loss of the integrated 162 kb viral genome, with accompanying telomere truncation in the latter instance, may affect essential telomere functions and play a role in iciHHV-6-associated disease (11, 28).

A global iciHHV-6 prevalence of ~1% is frequently quoted (6, 11, 13, 29); however, most prevalence studies have been small or included selected patient groups, leading to imprecise estimates. In addition, many populations have not been investigated. In the most detailed large study to date, Gravel et al. (30) screened 19,597 participants from the CARTaGENE study, a population-based cohort from the Canadian province of Quebec, and reported an overall iciHHV-6 prevalence of 0.58% (30); iciHHV-6A and iciHHV-6B prevalence were 0.23% and 0.34%, respectively (30). The overall iciHHV-6 prevalence was higher in several smaller studies in the UK (0.8%–1.9%) (31–34) and Czech Republic (0.95%–1.47%) (35, 36), whereas iciHHV-6 prevalence was similar or lower in studies from Japan and China (0.21%–0.6%) (37–40). Overall, these results suggest that there is significant geographical variation in iciHHV-6 prevalence.

It has been proposed that much of the iciHHV-6 detected today is a consequence of viral integration events that occurred thousands of years ago (34, 41). Although iciHHV-6 genomes are highly conserved, phylogenetic analysis of viral genome sequences reveals clades of nearly identical genomes (34, 41–43). Data from fluorescent *in situ* hybridization (FISH) studies and analysis of the HHV-6 genome integration site in telomeres suggest that iciHHV-6 genomes within individual clades share an integration site (34, 41, 44). This is consistent with descent from a common ancestor; thus, these genomes represent a single ancestral iciHHV-6 lineage. In individuals of European ancestry, the common lineages include an HHV-6A clade A2 integration in chromosome 17p, an HHV-6B clade B8 integration in chromosome 17p, and an HHV-6B clade B6 integration in chromosome 9q (3, 10, 34, 41, 44). By contrast, in Japan and China, ancestral integrations of iciHHV-6A and iciHHV-6B in chromosome 22q predominate, with additional integrations in Yp, Xp, and 17q (38, 39, 45). Understanding the prevalence of ancestral viral lineages in different countries and regions is essential, as disease associations may vary by chromosomal integration site or viral lineage.

Studies on disease associations with iciHHV-6 have generally been small and focused on specific diseases, making it difficult to draw firm conclusions on the overall impact of iciHHV-6 on health; however, a meta-analysis suggested that iciHHV-6 was more frequent in hospitalized individuals compared to controls (17). An association with systemic lupus erythematosus was recently reported in two cohorts (40). Analysis of the CARTaGENE study revealed an unexpected association with angina pectoris, providing the first evidence that iciHHV-6 may contribute to the risk of a common condition (30).

The overall aim of this study was to understand the clinical impact of iciHHV-6. However, a more comprehensive understanding of the epidemiology of iciHHV-6 was needed to design robust disease association studies. The population- and family-based Generation Scotland: Scottish Family Health Study (GS:SFHS) cohort of approximately 24,000 participants was used to investigate iciHHV-6 prevalence and phenotypic associations in the UK and to follow iciHHV-6 inheritance within family pedigrees. In addition, approximately 8,000 females were analyzed from the Breakthrough Generations Study (BGS, now renamed the Generations Study and funded by Breast Cancer Now), a cohort study of women from the UK general population (46). We show that iciHHV-6B prevalence varies significantly within the UK, iciHHV-6 is inherited in a normal fashion without detectable selection against iciHHV-6 genomes, and most iciHHV-6 in the UK is derived from nine ancestral integration events. We also, independently, observe the previously reported association with angina.

## RESULTS

### iciHHV-6 prevalence

The GS:SFHS cohort (47) (*n* = 23,930; Table 1 and Table S1) and 8,030 individuals from BGS (46) (Table S2) were screened for iciHHV-6 using qPCR and confirmatory droplet digital PCR (ddPCR) (48) (Table S3, Table S4). In GS:SFHS, 647 (2.74%) of the 23,637 DNA samples that fulfilled QC criteria were iciHHV-6-positive; 44 (0.19%) DNA samples were iciHHV-6A-positive, and 603 (2.55%) DNA samples were iciHHV-6B-positive (Table 1). Three individuals had two integrated HHV-6B genomes; therefore, 606 iciHHV-6B genomes were detected. There was no significant variation in the prevalence of iciHHV-6-positive individuals by age or sex, overall or following stratification by viral species (Table 1).

**TABLE 1:** Characteristics of iciHHV-6-positive GS:SFHS participants

Demographic	All, no.	iciHHV-6-positive (A or B), no. (%^*a*^) or *P* value	iciHHV-6A-positive, no. (%^*a*^) or *P* value	iciHHV-6B-positive, no. (%^*a*^) or *P* value
Participants	23,637	647 (2.74)	44 (0.19)	603 (2.55)
Families	6,913	368	25^*a*^	345^*a*^
Sex				
Male	9,743	272 (2.79)	15 (0.15)	257 (2.64)
Female	13,894	375 (2.70)	29 (0.21)	346 (2.49)
*P* value		0.72	0.28	0.50
Age group (years)				
Missing	5	1	1	
18–24	2,128	54 (2.54)	5 (0.23)	49 (2.30)
25–34	3,204	91 (2.84)	8 (0.25)	83 (2.59)
35–44	4,341	132 (3.04)	5 (0.12)	127 (2.93)
45–54	5,120	135 (2.64)	5 (0.10)	130 (2.54)
55–64	5,972	149 (2.49)	14 (0.23)	135 (2.26)
65–74	2,075	65 (3.13)	5 (0.24)	60 (2.89)
≥75	792	20 (2.53)	1 (0.13)	19 (2.40)
*P* value		0.56	0.41	0.43
Nationality				
Scottish	19,807	577 (2.91)	35 (0.18)	542 (2.74)
English	1,527	19 (1.24)	6 (0.39)	13 (0.85)
*P* value		<0.0001	0.07	<0.0001

^*a*^ Percentages shown are row percentages, i.e., iciHHV-6 prevalence within subcategory; two extended families included individuals who were iciHHV-6Apositive or iciHHV-6B-positive; therefore, the sum of iciHHV-6A and iciHHV-6B families exceeds the total number of iciHHV-6-positive families. Further information on nationality is provided in Table S5. *P* values were calculated using Fisher’s exact tests.

In BGS, 90 of 7,910 samples that fulfilled QC criteria were iciHHV-6-positive (1.14%); 20 samples were iciHHV-6A-positive (0.25%), and 70 samples were iciHHV-6B-positive (0.88%). One individual had two iciHHV-6B genomes. There was no difference in overall iciHHV-6 positivity, or iciHHV-6A or iciHHV-6B positivity, between incident breast cancer cases from the cohort and controls without breast cancer from the same cohort (Table S2); the two groups of participants were therefore considered together in the analyses of regional differences in prevalence described below.

### Viral genome composition

ddPCR results suggested that most iciHHV-6-positive individuals had a single genome with one U region and two DRs (U_1_DR_2_) in each cell, and four had suggestive evidence of two iciHHV-6B genomes (U_2_DR_4_; Table S4). More unusual compositions were observed in 39 individuals in GS:SFHS and 12 in BGS; genomes consisting of a single DR (DR_1_-only) were most common, followed by genomes with two U regions and three DRs (U_2_DR_3_; Table S4).

### Variation in iciHHV-6 prevalence within the UK

**Fig 1:**
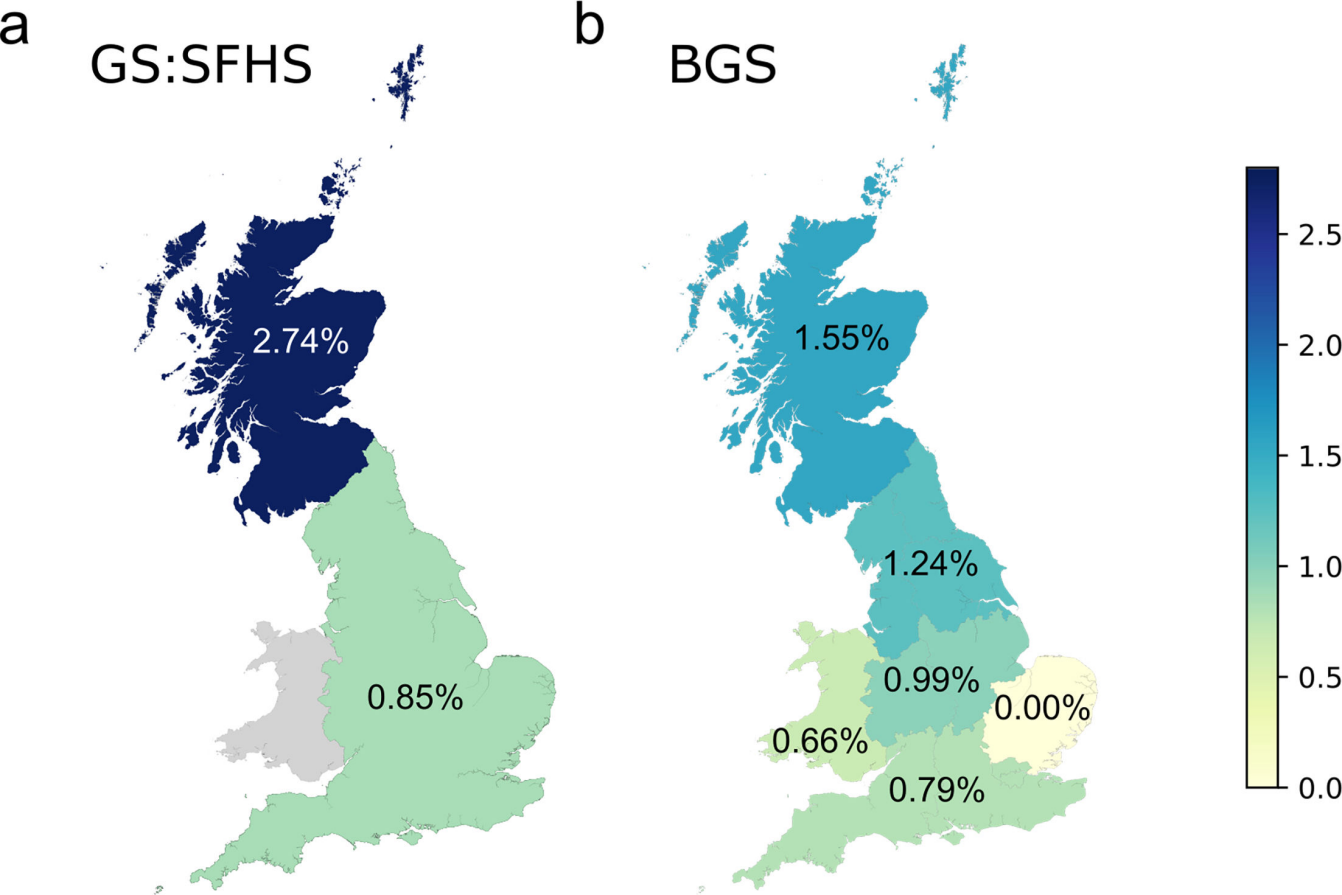
Prevalence of iciHHV-6B in the UK. (a) Prevalence of iciHHV-6B in GS:SFHS by self-reported nationality. (b) Prevalence of iciHHV-6B in BGS by region of residence: Scotland; Northern England; Central England; Southern England; East Anglia; and Wales. Map source: Office for National Statistics licensed under the Open Government License v.3.0.

GS:SFHS participants who self-identified as Scottish were more likely to be iciHHV-6B-positive than those who self-identified as English (2.74% vs 0.85%, respectively, *P* < 0.0001; Table 1; Fig. 1). Differences by self-identified nationality were supported by analysis of participant birthplace and parental nationality (Table S5). English GS:SFHS participants were more likely to be iciHHV-6A-positive than Scottish participants, but the differences were not statistically significant (*P* = 0.07; Table 1). Too few individuals had a nationality other than Scottish or English for meaningful analysis (Table S5).

The BGS cohort was used to investigate further the differences in iciHHV-6B prevalence in mainland Britain using data on country (Scotland, England) and region of residence (northern, central, and southern England, and East Anglia) rather than nationality. The iciHHV-6B prevalence was 1.55% (7/453) in Scotland, 1.24% (18/1,453) in the north of England, 0.99% (13/1,313) in central England, and 0.79% (30/3,783) in the south of England (Fig. 1). None of the 562 participants resident in East Anglia was iciHHV-6-positive. Heterogeneity by region was statistically significant (*P* = 0.022).

### iciHHV-6 is associated with an increased risk of angina

**Fig 2:**
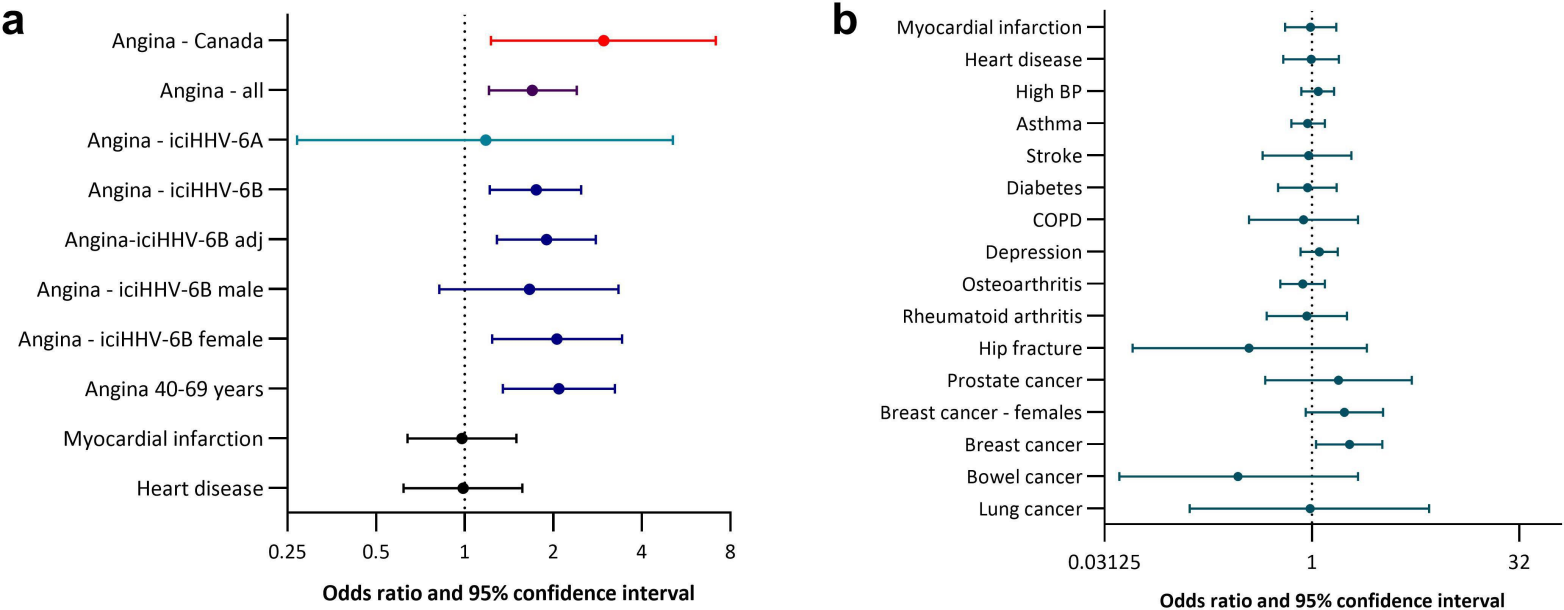
Association between iciHHV-6 and diseases forest plots showing OR and 95% CIs for risk of angina and other conditions with data available at baseline (date of recruitment). (a) Association between iciHHV-6B and angina. Angina - Canada: the adjusted OR and 95% CIs from the CARTaGENE study (30) are shown for comparison. Angina - all: iciHHV-6A and iciHHV-6B combined, adjusted for age and sex. Angina - iciHHV-6A and Angina - iciHHV-6B: data by viral species, adjusted for age and sex. Angina - iciHHV-6B adj: analysis adjusted for age, sex, body mass index, average systolic blood pressure, body fat, nationality, Scottish Index of Multiple Deprivation (quintiles), and smoking status. Angina - iciHHV-6B male, Angina - iciHHV-6B female, and Angina 40–69 years (the age range in the CARTaGENE study) are adjusted for the variables included in iciHHV-6B adj. Adjustments for age include age as a continuous variable. (b) Association between iciHHV-6 and 15 conditions with baseline data. COPD, chronic obstructive pulmonary disease. Data for Parkinson’s disease are not shown as there were no iciHHV-6-positive individuals with Parkinson’s disease.

Data collected using the validated WHO Rose angina questionnaire (49), which were available from 94.7% of participants in the GS:SFHS (47), were used to assess whether iciHHV-6 is associated with angina pectoris, as previously reported (30). A significant association was detected between iciHHV-6 status and angina in unadjusted analysis and following adjustment for age and sex (odds ratio [OR] = 1.7; 95% confidence interval [CI] 1.21, 2.41; Table 2; Fig. 2). There were only two iciHHV-6A-positive individuals with angina, and the association with iciHHV-6A was not statistically significant (OR = 1.18; 95% CI 0.27, 5.11; Fig. 2); further analyses therefore focused on iciHHV-6B. A significant association with iciHHV-6B remained following the inclusion of known risk factors for angina. Following adjustment for age, sex, body mass index, percentage body fat, average systolic blood pressure, Scottish Index of Multiple Deprivation (in quintiles), smoking status, and nationality, the OR for angina in iciHHV-6B-positive participants was 1.91 (95% CI 1.29, 2.79; Fig. 2, Table S6). In analyses stratified by sex, the risk was slightly higher for females (OR = 2.06; 95% CI 1.24, 3.43) than males (OR = 1.66; 95% CI 0.82, 3.34), but differences by sex were not statistically significant. Participants in the age range of the CARTaGENE study (40–69 years), in which the association was first reported (30), had a slightly higher OR than the overall study population (OR = 2.09; 95% CI 1.35, 3.24).

**TABLE 2:** Association between iciHHV-6B and angina

Angina	All *N* (%)	iciHHV-6-negative *N* (%)	iciHHV-6-positive (A or B) *N* (%)	iciHHV-6A-positive *N* (%)	iciHHV-6B-positive *N* (%)
No	21,462 (95.9%)	20,897 (96.0%)	565 (93.5%)	38 (95.0%)	527 (93.4%)
Yes	917 (4.1%)	878 (4.0%)	39 (6.5%)	2 (5.0%)	37 (6.6%)
OR^*a*^ (95% CI)			1.7 (1.21, 2.41)	1.18 (0.27, 5.11)	1.75 (1.22, 2.49)

^*a*^ OR following adjustment for age and sex.

### Exploratory association analysis of other phenotypes and iciHHV-6

Questionnaire data, anthropometric measurements, and blood test results from the GS:SFHS research clinic visit were compared in iciHHV-6-positive and iciHHV-6-negative individuals, with stratification by HHV-6A and HHV-6B (Table S6). Females with iciHHV-6, or iciHHV-6B, were shorter in height than iciHHV-6-negative participants (*P* = 0.02 and *P* = 0.007, respectively); although differences were statistically significant and persisted after adjustment for nationality, the effect size was small. Similar differences were not detected in male GS:SFHS participants, and differences in height by iciHHV-6 status were not confirmed in the BGS, suggesting that this difference is unlikely to be genuine.

### Exploratory analysis of the association between self-reported conditions and iciHHV-6

The GS:SFHS questionnaire elicited information about 15 medical conditions, including breast cancer. Self-reported breast cancer showed a significant association with iciHHV-6 when individuals of both sexes were analyzed (OR = 1.88; 95% CI 1.01, 3.52, *P* = 0.048). There was one male participant who self-reported breast cancer, but the diagnosis was not validated, and significance was lost when the analysis was restricted to females (OR = 1.72; 95% CI 0.9, 3.29, *P* = 0.10; Fig. 2). The initial observation prompted investigation of the association with breast cancer using samples from women with breast cancer and women without this disease from the BGS cohort. Consistent with a previous study by Gravel et al., 2020, and as mentioned above, the association with breast cancer was not confirmed in BGS (OR = 1.12; 95% CI 0.70, 1.79; Table S2) (50).

No clear associations with the remaining 14 conditions included in the GS:SFHS questionnaire were detected (Fig. 2, Table S7). There was no evidence of an increased risk of myocardial infarction (MI) or self-reported heart disease in iciHHV-6-positive individuals (Fig. 2).

### iciHHV-6 inheritance

GS:SFHS is family based, and this allowed the inheritance of iciHHV-6 to be studied. Overall, 368/6,913 GS:SFHS pedigrees included iciHHV-6-positive participants; 25 pedigrees included iciHHV-6A-positive individuals, and 345 pedigrees had iciHHV-6B-positive individuals. Two pedigrees included both iciHHV-6A-positive and iciHHV-6B-positive individuals (Fig. S1). Five pedigrees had two separate introductions of iciHHV-6B, and a further two included individuals with two integrated viruses; therefore, there were 377 separate iciHHV-6 introductions into the 6913 GS:SFHS families.

There were 442 participants with an iciHHV-6-positive family member. Of the 338 participants with an iciHHV-6-positive parent, 172 (50.89%) were iciHHV-6-positive, consistent with a normal pattern of inheritance (Table S8). All iciHHV-6-positive individuals within informative pedigrees had a positive parent; thus, there was no evidence of *de novo* iciHHV-6 lineages within the study population. There was no difference in the frequency of inheritance by virus type (HHV-6A or HHV-6B) or by sex of the parent (Table S8).

Data from BGS were used to examine parity and age at birth of a first child by iciHHV-6 status, as the design of the GS:SFHS was not appropriate for this analysis. There was no evidence that iciHHV-6 positivity, overall or following stratification by HHV-6 species, was associated with fewer children, fewer pregnancies, or older age at first birth (Table S9).

### Ancestral iciHHV-6A and iciHHV-6B lineages in GS:SFHS

We developed a panel of complementary assays to determine the ancestral viral lineage of the iciHHV-6 genomes. PCR amplification of the virus-host junction fragment at the integration site, followed by sizing or sequencing of the amplicon, was considered the definitive method of assigning viral lineage and was achieved for 402/650 (62%) of the iciHHV-6 genomes (Fig. S3 and see below). Viral clade assignment and identification of linked host haplotypes were also used to predict viral lineage and identify the chromosome end involved. For most viral genomes, clades were assigned using custom genotyping assays that detect clade B4-, B5-, B6-, and B8-specific SNPs (41) (Table S3). Clade assignments were validated using phylogenetic analysis of 16 iciHHV-6 genomes sequenced as part of the current study and five GS:SFHS iciHHV-6 genome sequences available from a previous study (34).

**Fig 3:**
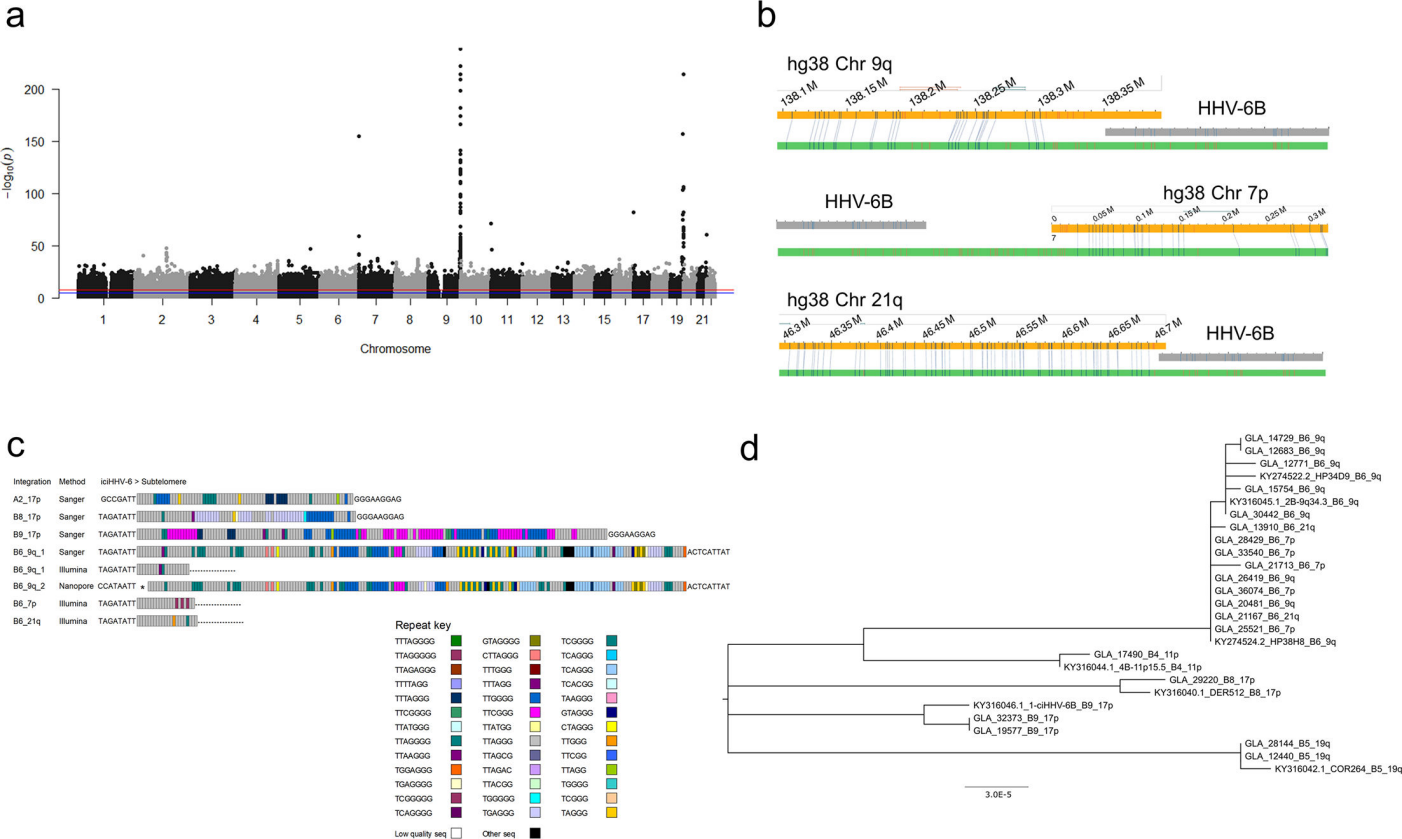
HHV-6 integrations and ancestral viral lineages. (a) Manhattan plot showing an association between iciHHV-6B and imputed genetic variants close to the telomeres of chromosome 7p, 9q, 11p, 17p, and 19q. (b) Analysis of samples with clade B6 iciHHV-6B genomes by optical genome mapping. Consensus maps (cmaps, green bars) from optical genome mapping were assembled using the human genome (hg38 build, yellow bar) as a reference on the basis of fluorescent labeling of CTTAAG recognition sites that aligned to the human reference (blue vertical lines) or did not align to the human reference (red vertical lines). The pattern of fluorescent labels unique to the HHV-6B genome (gray bar) could be identified in unaligned fluorescent labels at the ends of cmaps that aligned to chromosome 7 p, 9q, and 21q for GLA_21713, GLA_32597, and GLA_13910, respectively. (c) Patterns of telomere and telomere-like repeats in the junction fragment between iciHHV-6 and low copy-number sequence in the subtelomere. Each small rectangle represents a telomere or telomere-like sequence as indicated in the Repeat key. * Denotes an omitted telomere-repeat array of 290 hexamers, 65% TTAGGG interspersed with telomere-likes repeat, almost entirely TTAGAC-CTAGGG dimers. B6_9q_1 refers to B6_9q_hap1 (B6_9q_T2), B6_9q_2 refers to B6_9q_hap2 (B6_9q_T1). (d) Evolutionary analysis by Maximum Likelihood method of 20 iciHHV-6B genome sequences from GS:SFHS and seven publicly available iciHHV-6B genome sequences. Branch tips are labeled with sample name and lineage, where known. The evolutionary history was inferred using the Maximum Likelihood method and Tamura-Nei model (51). Branch lengths are measured in the number of substitutions per site. There were a total of 140,246 positions in the final data set.

Imputed and phased genome-wide genotyping data were available for 36 iciHHV-6A-positive, 525 iciHHV-6B-positive, and 19,406 iciHHV-6-negative participants. A genome-wide association study comparing iciHHV-6A-positive and iciHHV-6-negative individuals did not reveal any genome-wide significant associations. However, for iciHHV-6B, there were highly significant associations at the ends of chromosome arms 7p, 9q, 11p, 17p, and 19q (Fig. 3A); subsequent analyses led to the identification of iciHHV-6B-associated host haplotypes at these chromosome ends. Further genome-wide association analysis following the division of the participants into groups based on host haplotype led to the identification of an additional haplotype at the end of chromosome 21q (Fig. S2 and S3). The identified haplotypes were strongly associated with, and in some cases exclusive to, iciHHV-6B-positive individuals (Table S10). The strength of these associations (Table S10), plus their mutual exclusivity (Fig. S2), suggested that these host haplotypes are in linkage disequilibrium with the integrated virus.

**Fig 4:**
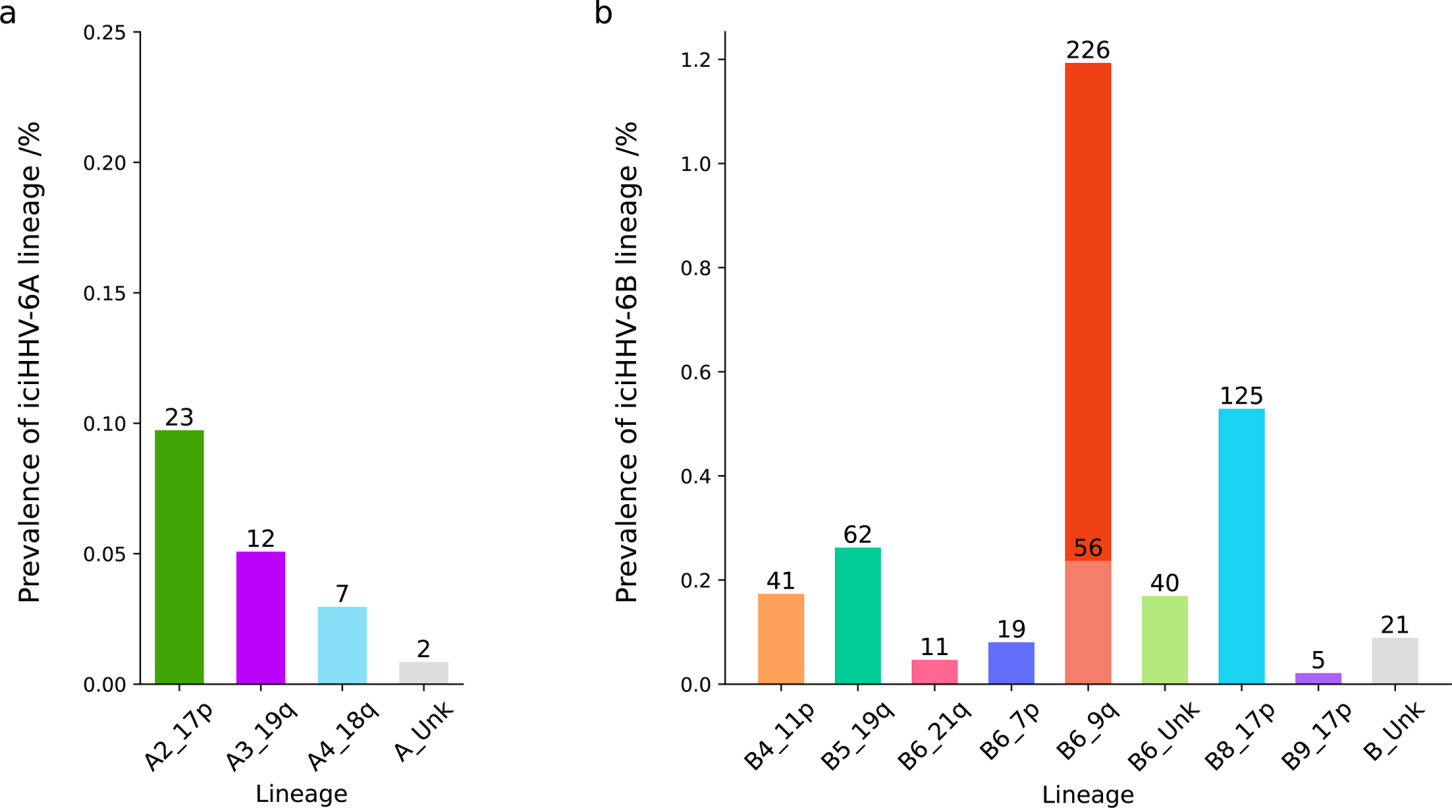
Proportions of the common ancestral viral lineages within GS:SFHS. A panel of complementary assays was used to assign viral lineage to the 44 iciHHV-6A and 606 iciHHV-6B genomes detected in GS:SFHS. (a) iciHHV-6A. (b) iciHHV-6B.

### Clade B6 genomes

iciHHV-6 genotyping identified 335 genomes in clade B6, a clade which has been associated previously with chromosome 9q integration (34, 41). Host haplotype analysis identified two distinct haplotypes at the end of 9q that were associated with clade B6 iciHHV-6B genomes (B6_9q_hap1, *n* = 189, and B6_9q_hap2, *n* = 56). Additional haplotypes associated with B6 genomes were identified at the ends of 7p and 21q. To date, only one viral genome integration site has been identified in each phylogenetic clade; therefore, further analyses were carried out to explore whether these four different haplotypes represent distinct ancestral HHV-6B integration events.

The iciHHV-6B-positive 2B-9q34.3 cell line has a clade B6 genome (34, 41), and the viral genome has been shown to be integrated into chromosome 9q by FISH (3). We confirmed this location using targeted locus amplification (TLA) and optical genome mapping (OGM), a technique that has been used previously to identify iciHHV-6 integration sites (52) (Fig. 3b, Fig. S4). To characterize the 9q integration site, we performed long-read (Nanopore) sequencing of 2B-9q34.3 genomic DNA. This generated 464,000 reads with an average N50 of 8.7 kb that included a single 8.4 kb read extending from the viral DR_R_, into the host DNA sequence, through the telomere repeat array. This sequence read enabled the design of a primer (9q_TJ1, Table S3) that, when paired with a DR primer, amplified a 1.6 kb fragment containing the virus-host junction in the 2B-9q34.3 cell line (Fig. 3c, Table S3) and in samples from 206 participants with B6_9q_hap1. This fragment was not amplified in any of the 18 tested samples from individuals with B6_9q_hap2. Nanopore sequencing of high molecular weight DNA from two GS:SFHS participants with B6_9q_hap2 generated a total of 1.7 million and 2.0 million reads with an N50 of 18.1 kb and 7.1 kb, respectively. Sequence analysis of both samples identified a rearranged HHV-6B viral genome with two tandem DRs at the telomeric end of the viral genome and integration into host DNA at DR_R_-T1 rather than DR_R_-T2 (Fig. S5). The virus-host junction was subsequently PCR amplified in a 4.6 kb fragment in 45/50 samples from individuals with B6_9q_hap2 using a U100 primer paired with 9q_TJ1 (Fig. S5). Although the length of the telomere-repeat array at the virus-host junction differed between individuals with B6_9q_hap1 and individuals with B6_9q_hap2, the pattern of telomere and telomere-like repeats was consistent, strongly suggesting that all these individuals share a common ancestor (Fig. 3C). These lineages have been designated B6_9q_T2 (T2 adjacent to integration site, associated with B6_9q_hap1) and B6_9q_T1 (T1 adjacent to integration site, associated with B6_9q_hap2).

HHV-6 WGS and phylogenetic analysis of five genomes associated with B6_9q_hap1 and two with B6_9q_hap2 confirmed clustering in the B6 clade (Fig. 3D). However, a C > T variant at position 42,889 in the HHV-6B reference genome was identified in the majority (185/202, 91.6%) of B6_9q_T2 genomes. HHV-6B B6 genomes with this variant clustered together within clade B6. All B6_9q_T1 genomes (53/53 tested) had the C variant. Overall, 282 (46.5%) of the 606 iciHHV-6B genomes in GS:SFHS had viral genomes assigned to the B6_9q ancestral lineage: 226 (37.3%) to B6_9q_T2 and 56 (9.2%) to B6_9q_T1 (Fig. 3 and 4, and Fig. S3).

Host haplotype analysis identified 16 iciHHV-6B-positive individuals from 12 families with a 7p haplotype; all had the viral clade B6-specific SNP, and all tested samples (12/12) had the C variant at position 42,889. A clade B6-associated haplotype was also identified at the end of chromosome 21q in 10 individuals with iciHHV-6B (Fig. S2 and Table S10). All genomes associated with this haplotype had the C variant at position 42,889. HHV-6 WGS and phylogenetic analysis performed on two viral genomes from each of these groups confirmed inclusion in the B6 clade (Fig. 3D). HHV-6 WGS reads extending from the DR_R_ into the telomere repeat arrays enabled analysis of the pattern of perfect and imperfect telomere repeats at the viral end of the virus-host junction fragment in these sequenced samples (Fig. 3C). A pattern was shared between the two genomes with the 7p haplotype, and a different pattern was shared between the two genomes with the 21q haplotype. These were distinct from the patterns identified in samples with viral genomes integrated into chromosome 9q. OGM confirmed viral integrations into the short arm of chromosome 7 and the long arm of chromosome 21 (Fig. 3b and Fig. S4). Based on haplotype and pedigree analysis, 19 (3.1%) of the iciHHV-6B genomes in GS:SFHS were classified in the B6_7 p lineage and 11 (1.8%) in the B6_21q lineage (Fig. 4).

Forty GS:SFHS participants, including 14 lacking host genetic data, had evidence of a B6 clade virus from viral SNP genotyping or pedigree analysis, but the ancestral viral lineage was not determined.

### Clade B8 and B9 genomes

Testing of available DNA from 569 iciHHV-6-positive individuals, using a previously described 17p integration site PCR assay and Sanger sequencing, identified a 1.5 kb junction fragment in 122 (22%) iciHHV-6B-positive samples (Fig. S3). The pattern of telomere and telomere-like repeats in the sequenced junction fragments (*n* = 122) was highly similar among iciHHV-6B-positive individuals (Fig. 3C) and to the integration site fragment previously sequenced from clade B8 genomes (10, 34, 53). Three individuals who lacked satisfactory 17p integration site analysis had genomes classified in B8_17p based on haplotype analysis (Fig. S3) or clade assignment based on either the presence of the clade B8-specific SNP or the clade B8-specific sequence of the proximal variable region of T1 (9). Overall, 125 (0.53%) GS:SFHS participants had iciHHV-6B genomes classified in B8_17p (Fig. 4 and Fig. S3).

Using the same primer pair, a larger 2 kb junction fragment was amplified from five iciHHV-6B-positive individuals from two GS:SFHS families. Sanger sequencing showed that the pattern of telomere and telomere-like repeats was the same as that in the 1-ciHHV-6B cell line (Fig. 3C) (9, 34), which has a 17p integration demonstrated by FISH (3) and TLA (Fig. S4). HHV-6 WGS analysis of a sample from each of the two GS:SFHS families identified HHV-6B genomes that cluster with 1-ciHHV-6B on the HHV-6B phylogenetic tree. We have named this clade B9 and the respective lineage B9_17p (Fig. 4).

### Clade B5 genomes

Clade B5-specific SNP genotyping or possession of an iciHHV-6B-associated chromosome 19q haplotype (Fig. 4;Fig. S3 and Table S10) identified 62 iciHHV-6B genomes (10.2%) that we have classified in the B5_19q ancestral viral lineage. HHV-6 WGS of two samples in this group confirmed inclusion in clade B5 (Fig. 3D).

### Clade B4 genomes

Clade B4-specific SNP genotyping or possession of an iciHHV-6B-associated chromosome 11p haplotype (Fig. S3 and Table S10) identified 41 iciHHV-6B (6.8%) genomes that we have classified in the B4_11p ancestral lineage (Fig. 4 ; Fig. S3).

### Ancestral lineages of iciHHV-6A

Assignment of iciHHV-6A genomes to ancestral viral lineages was performed using the chromosome 17p integration site assay, HHV-6 WGS, DR_R_-T1 length, and host haplotype analysis. However, since there were no significant genome-wide associations for iciHHV-6A, the haplotype data should be interpreted with caution. Using the 17p integration site assay (Table S3), amplicons with an estimated size of 1.5 kb were detected in samples from 23/40 iciHHV-6A-positive individuals from 10/22 GS:SFHS families. Sanger sequencing of the virus-host junction fragment from these 23 individuals confirmed that all shared a highly similar sequence that has been associated with clade A2 viruses (A2_17p lineage; Fig. 3C) (2, 10, 44). Two related individuals in GS:SFHS and one BGS participant carried an A2_17p lineage genome with a single DR and a partially deleted U region. HHV-6 WGS of the viral genome from one of the GS:SFHS participants showed that the viral genome was deleted from the 5′-end to position 146,988 relative to U1102 (GenBank accession: X83413.2) with no evidence of telomere repeats in the reference viral sequence at the point of the deletion (U_partial_DR_1_; Fig. S5).

Grouping of the remaining iciHHV-6A-positive samples, based on the length of a DR_R_-T1-containing amplicon, led to the identification of host haplotypes associated with clade A4 genomes integrated in chromosome 18q (A4_18q) and clade A3 genomes integrated in chromosome 19q (A3_19q). Based on host haplotype and DR_R_-T1 length, 12 individuals from seven families were provisionally assigned to A3_19q and seven individuals from six families to A4_18q. In one father-daughter pair, the A4_18q haplotype was identified in the father, and a recombined haplotype, sharing the most telomeric portion of the associated 18q haplotype, was detected in the daughter.

### Genomes with unusual compositions are stably inherited and arise following integration

Almost all genomes with unusual genome compositions (DR_1_-only, U_2_DR_3_, DR_2_-only, and U_partial_DR_1_) belonged to the common ancestral lineages described above, indicating that deletion or duplication events occurred following integration. All iciHHV-6A genomes with extra DRs belonged to the A4_18q lineage, and all iciHHV-6A genomes in this lineage had extra copies of DR. Genomes with unusual compositions were stably inherited within pedigrees (Fig. S1). We identified a sibling pair in whom one individual had a complete copy of the HHV-6B genome, while the other had a DR_1_-only genome. Both had the B6_9q_hap1 haplotype and the B6_9q_T2 junction fragment, suggesting that a deletion event had occurred either in a germ cell of the affected parent or at the conception of the sibling with the DR-only genome.

## DISCUSSION

To our knowledge, this is the largest study of iciHHV-6 that includes details on lineage. The results provide further evidence that iciHHV-6 genomes are common and may contribute to disease risk. We screened almost 32,000 individuals in the UK for iciHHV-6A and iciHHV-6B and analyzed prevalence, genome composition, phenotype and disease associations, inheritance, and ancestral viral lineages. We show an unexpectedly high prevalence of iciHHV-6B in Scottish people and significant regional variation in prevalence within the UK. This marked variation in prevalence over relatively short distances has important implications for the design of iciHHV-6-disease association studies. The previously described association with angina was replicated, providing compelling evidence that iciHHV-6 is not innocuous. Through detailed analysis using complementary methods, we identified the ancestral viral lineage of the majority of iciHHV-6 genomes and showed that most iciHHV-6 in the UK results from nine ancient viral integrations. The results are relevant to genetics and virology as well as clinical medicine.

To determine iciHHV-6 status, samples from participants in the GS:SFHS and BGS were initially screened by qPCR, and then secondary analyses were performed using multiple ddPCR assays. We targeted sequences in the viral DR in the initial screen to avoid missing iciHHV-6 genomes that lack viral U region sequences. Although ddPCR provides extremely accurate estimates of the mean number of viral targets per cell, with results close to integer values being highly suggestive of iciHHV-6, this technique does not provide direct evidence of integration. In the GS:SFHS, further evidence of germline integration of HHV-6 genomes was obtained for 554 of the 647 iciHHV-6-positive participants through analysis of family pedigrees (*n* = 442), direct amplification of the junction fragment at the viral integration site (*n* = 402), or both (*n* = 290).

The overall prevalence of iciHHV-6 in GS:SFHS was 2.74% (647/23,637), comprising 0.19% with iciHHV-6A and 2.55% with iciHHV-6B. To our knowledge, this is the highest prevalence reported in any large study of iciHHV-6 to date. Although most participants in GS:SFHS were Scottish, the inclusion of extended families resulted in participants from other countries and ethnicities. There was a striking difference in iciHHV-6B positivity between individuals self-identifying as Scottish compared to those identifying as English, with a prevalence of 2.74% in Scottish and 0.85% in English participants (Fig. 1). Further analyses, which included data from BGS participants, showed significant regional variation in iciHHV-6B prevalence, with a higher prevalence in the north of mainland Britain (Fig. 1). The prevalence of iciHHV-6A was much lower and did not show statistically significant differences within the UK, although the proportion of iciHHV-6A-positive individuals was higher in participants self-identifying as English or living in England (Table S5). iciHHV-6A prevalence was similar to that reported from the CARTaGENE study in Quebec, Canada (30), but iciHHV-6B prevalence was higher in both Scotland and England than in Quebec. Although iciHHV-6 is a global phenomenon, this study provides evidence that prevalence varies significantly in different geographical areas, even over relatively small distances. Furthermore, the prevalence of iciHHV-6A and iciHHV-6B varies independently, and it is therefore important to consider each virus separately and not simply the proportion of people who have iciHHV-6A or iciHHV-6B. This finding presents challenges for studies investigating iciHHV-6 disease associations, as careful case-control matching and adjustment for region (birthplace or residence) and ethnicity are required to prevent misinterpretation of results and reporting of spurious associations.

Gravel et al. (30) reported an association between iciHHV-6 and angina in the first large study of iciHHV-6, with an OR for angina of 2.97 (95% CI, 1.23, 7.15) after adjustment for other risk factors (30). Here, we replicated this association in GS:SFHS. In unadjusted analyses, a significantly increased risk of angina was detected for iciHHV-6 overall and for iciHHV-6B with ORs of 1.66 (95% CI, 1.18, 2.32) and 1.69 (95% CI, 1.19, 2.33), respectively, with a non-significant increased risk in iciHHV-6A-positive participants. Adjustment for known risk factors for angina resulted in an increase in the OR, which was maintained following adjustment for Scottish nationality (OR = 1.91, 95% CI, 1.29, 2.79). The 95% confidence intervals for the association between iciHHV-6 and angina in GS:SFHS and the CARTaGENE study overlapped, and in both studies, the risk was non-significantly greater for females than males (30). Neither study found an association with MI (30). The similarity between the findings in the two largest independent studies of iciHHV-6 suggests that the association is real. The biological mechanism underlying the association with angina is not yet clear but has biological plausibility, given the association between atheromatous plaques and both inflammation and telomere shortening (54–57). The lack of an association with MI may simply reflect reduced power due to the lower numbers of individuals with MI in these studies. Angina is a symptom rather than a disease, and further studies are needed to shed light on the underlying pathology associated with this finding.

We did not find robust evidence of other phenotypic or disease associations in the preliminary analysis performed in this study. Follow-up studies will include a more in-depth analysis of disease associations through linkage with medical records, but, ultimately, given the relatively low frequency of iciHHV-6, much larger studies are needed to have the power to identify disease associations in an agnostic fashion. The lack of an association between iciHHV-6 and age (at least below the age of 75 years), the lack of selection against iciHHV-6-positive offspring, and the persistence of ancient integrations suggest that the effects of these lineages of iciHHV-6 are not catastrophic. However, iciHHV-6 can cause disease through viral reactivation (7) and may be associated with an increased risk of angina, thus suggesting that it is not a harmless phenomenon.

The family-based structure of the GS:SFHS allowed us to study the inheritance of iciHHV-6 within pedigrees. We confirm that iciHHV-6 is inherited in a normal fashion, with no evidence for selection for or against iciHHV-6-positive individuals within this study (Table S8). We also show that children with iciHHV-6 are equally likely to inherit the virus from their mother or their father (Table S8). Previous smaller studies have not detected significant differences in maternal and paternal transmission (58, 59), but Miura et al. (59) reported a non-significant excess of paternal transmission (59). Since HHV-6A has been associated with primary unexplained infertility (60), we used data from the BGS to compare the number of children born to iciHHV-6-positive vs iciHHV-6-negative mothers, with stratification by iciHHV-6A and iciHHV-6B. Although the numbers are relatively small, we found no evidence that iciHHV-6A or iciHHV-6B-positive mothers had fewer children than controls (Table S9).

In GS:SFHS pedigrees, we did not detect any genotyped individuals with *de novo* germline integration of either HHV-6A or HHV-6B. Instead, we found that a small number of ancient viral lineages account for most iciHHV-6 genomes in our study. Using complementary methods to analyze ancestral viral lineage, we predicted the lineage of 95% of the iciHHV-6A and almost 90% of the iciHHV-6B genomes in GS:SFHS. Most iciHHV-6A genomes belonged to three ancestral lineages (A2_17p, A3_19q, and A4_18q) that have been described previously (Fig. 4). The four most common ancestral lineages of iciHHV-6B, namely B6_9q, B8_17p, B5_19q, and B4_11p, accounted for 84% of the iciHHV-6B genomes in GS:SFHS (Fig. 4). The B6_9q lineage was identified in almost 1.2% of GS:SFHS study participants and is likely to account for some, although not all, of the excess of iciHHV-6B in the Scottish population. The marked differences in regional prevalence of iciHHV-6B in the UK may also result from dilution effects caused by more migration of iciHHV-6B-negative individuals into southern compared to northern regions. We cannot exclude the possibility that one or more ancestral iciHHV-6B lineages originated in Scotland.

We exploited the available genome-wide genotype data in the GS:SFHS to identify host haplotypes associated with iciHHV-6 and predict viral integration sites and ancestral lineages. Analysis of these iciHHV-6B-associated host haplotypes led to the identification of three different viral lineages, with integrations in chromosome 9q, 7p, and 21q, within the B6 clade. Our findings were unexpected in light of previously published data (41). To verify our results, we used OGM to confirm that the 7p and 21q haplotypes are associated with viral integration in 7p and 21q, respectively (Fig. 3B). We also analyzed the telomere repeats at the viral end of the integration site in the three different lineages and detected three different patterns of telomere and telomere-like repeats, consistent with independent integration events (Fig. 3C). Extremely short branch lengths characterize clade B6 on the phylogenetic tree (41), and we could not distinguish these lineages by phylogenetic analysis using HHV-6 WGS, although the C > T variant at position 42,889 did result in B6_9q_T2 genomes clustering together on the tree (Fig. 3D). Aswad et al. (41) associated clade B6 with integrations in 9q using FISH data from three individuals (41). FISH determines the chromosomal location of the integrated virus but is not suited to the analysis of large sample numbers; therefore, using FISH, the much less common clade B6 integrations in 7p and 21q integrations could easily be missed. There are several possible explanations for three related lineages within the B6 clade, which will be challenging to disentangle. Clade B6 viruses may have been circulating widely during a period when several viral germline integrations occurred. Alternatively, these B6 viruses may have been more likely to integrate and be maintained and less likely to be detrimental to the host than other lineages. It is also possible that an integrated B6 virus reactivated and reintegrated at a new location, either within an individual or following transmission of the virus to a new host.

The haplotype analysis of chromosome 9q led to the identification of a subgroup of individuals with an iciHHV-6B genome with tandem DRs at the 5′-end and integration at the viral T1. The junction fragment found in the T1 integration is very similar to that in the more common B6_9q_T2 integration. These results suggest that there is a single B6_9q ancestral lineage, but a rearrangement of the viral genome and recombination events in the associated host haplotype occurred after integration. Similarly, our data suggest that most of the unusual viral compositions arose following integration, as suggested by others (10, 61). While we cannot exclude the possibility that non-U_1_DR_2_ genomes integrated into the germline, structural rearrangements, possibly mediated by t-loops formed between the 3′ overhang of the iciHHV-6 associated telomere and telomere-repeat arrays within the viral genome, seem a more likely explanation. Other than A4_18q, the majority of iciHHV-6 genomes in all lineages are U_1_DR_2_, suggesting that this is the ancestral form.

The panel of assays that we developed to predict the ancestral viral lineage of iciHHV-6 genomes, including clade-specific genotyping and viral integration site assays, will be helpful to other researchers. Although haplotype analysis was invaluable in the present study, it is unclear how transferable the described haplotypes will be to other studies. We do not know whether the described haplotypes were present at the time of viral integration or whether they were inherited with the virus from a common ancestor in our study population. Host recombination events will limit the usefulness of this approach for the identification of viral lineages in all iciHHV-6-positive individuals. A recombination event in the host haplotype associated with the A4_18q lineage was identified in a father-daughter pair, and such events probably account for the absence of associated haplotypes in other individuals in this study. More recent integrations or introductions to a particular geographical locale will be more likely to share host haplotypes. In the present study, we detected a shared haplotype in 177/183 (97%) individuals with the B6_9q_T2 junction fragment and available genome-wide genotyping data, but a shared haplotype was detected in only 32/115 (28%) individuals with the B8_17p junction fragment and genome-wide genotyping data. These results are consistent with the idea that the 17p integration is older than the 9q integration (34, 41), although the timing of integrations is difficult to estimate.

The implications of iciHHV-6 extend beyond virology. As genome-wide association studies become larger and rare variants are interrogated, it is crucial that associations with ancient iciHHV-6 integrations are not misinterpreted as associations with host variants or haplotypes, and vice versa. iciHHV-6 genomes will be sequenced as part of whole-genome sequencing experiments, and iciHHV-6-positive individuals will be identified. It is important that we are able to provide affected individuals with reliable information about the consequences of iciHHV-6 positivity and how any harmful effects should be managed. Awareness and knowledge of iciHHV-6 in this age of high-throughput genomics are vital for affected individuals and scientists who stumble across these viral genomes.

## MATERIALS AND METHODS

### Study participants and samples

Samples and data from two studies were analyzed: the GS:SFHS (47, 62) and the BGS (46). GS:SFHS is a family-based cohort study that included 23,930 individuals aged 18–98 years at recruitment (https://genscot.ed.ac.uk/, Fig. S1). Most, but not all, participants were living in Scotland at enrollment; 83.6% self-identified as Scottish and 6.56% as English. Demographic details and history of 15 medical conditions were recorded for the participants, their parents, siblings, and grandparents using a pre-clinical questionnaire administered to the participants. The Rose angina questionnaire, a chest pain questionnaire, was used to determine whether participants suffered from angina (49). Physical measurements were recorded at a research clinic visit, and a blood sample was collected. Participants unable to attend the research clinic provided a saliva sample. DNA samples from 23,930 GS:SFHS participants were analyzed; 21,837 (89.4%) were extracted from blood and 2,543 (10.6%) from saliva.

The BGS is a UK-wide cohort study of women aged >16 years in the UK, focused on risk factors for breast cancer (46) (https://www.breakthroughgenerations.org.uk/home, now called the Generations study and funded by Breast Cancer Now, Table S2). BGS enabled a more detailed analysis of prevalence in different regions of England, reproductive factors, and breast cancer risk. Questionnaire data relating to lifestyle, reproductive factors, and breast cancer risk were collected along with a blood sample for DNA extraction. A subset of 8,030 participants, including 4,039 breast cancer cases and 3,991 controls, was tested for iciHHV-6. Cases residing in different regions of the UK were selected for inclusion and then matched with controls by age and area of residence; 90% of the selected participants were resident in England.

### Determination of iciHHV-6 status

Screening of the GS:SFHS cohort (*n* = 23,930; Table 1 and Table S1) and 8,030 individuals from BGS was performed using an HHV-6 DR1 TaqMan qPCR assay duplexed with a human β-globin gene assay (48) (Table S3). qPCRs were performed in a final volume of 12.5 µL and included 100 ng of template DNA, 1× TaqMan Universal MasterMix (Thermo Fisher Scientific, Paisley, UK), DR1 primers and probes at 300 nM and 200 nM, respectively, and β-globin primers and probe at 50 nM and 200 nM, respectively. DNA samples extracted from iciHHV-6A-positive (7A-17p13.3) and iciHHV-6B-positive (2B-9q34.3) lymphoblastoid cell lines (3) were used as positive controls, and DNA from iciHHV-6-negative cell lines and water were used as negative and no-template controls. Thermal cycling and data collection were performed on a QuantStudio 12K Flex Real-Time PCR System (GS:SFHS, Thermo Fisher Scientific) or a 7900HT Real-Time PCR system (BGS, Thermo Fisher Scientific); thermal cycling conditions were 50°C for 2 min, 95°C for 10 min, followed by 40 cycles of 95°C for 15 s and 60°C for 60 s. Samples with a C_T_ ≥ 32 in the β-globin component of the assay and samples with abnormal amplification plots failed quality control and were excluded from the study. Samples with a ΔC_T_ (C_T_DR1 – C_T_β-globin) value of less than 9 were selected for further analysis.

The selected samples were assayed using an HHV-6 U7 (63), an HHV-6A-specific DR6A, and an HHV-6B-specific DR6B ddPCR assay (Table S3). All viral ddPCR assays were duplexed with the human RPP30 assay (Bio-Rad Laboratories Ltd., Hertfordshire, UK). Reactions were performed in a final volume of 25 µL and contained 80 ng of DNA; viral primers and probes at 300 nM and 200 nM, respectively; 0.5× RPP30 primer and probe mix (Bio-Rad Laboratories Ltd.); 1× ddPCR SuperMix for probes without dUTP (Bio-Rad Laboratories); and 1 µL of a “restriction digestion mix” containing 5U of AluI in 1× CutSmart Buffer (New England Biolabs Ltd, Hertfordshire, UK). Droplet generation, thermal cycling, and data analysis were performed as described previously (33). ddPCR allowed precise and accurate quantification of the mcpc of the viral targets and typing of the viral species. Samples with a U7 mcpc >0.75, or DR1 mcpc >0.75 in the case of DR_1_-only genomes, were considered iciHHV-6 positive.

GS:SFHS samples were also analyzed with qPCR assays detecting the HHV-6A and HHV-6B POL genes, as described previously (64) (Table S3). Samples that were positive for HHV-6 DR1 and either DR6A or DR6B but negative for U7 and POL were analyzed using ddPCR assays for U100A or U100B as appropriate (Table S3). Viral composition (number of U and DR regions) was determined based on the mcpc of the DR6 and U7 targets in the ddPCR assays.

### Analysis of disease associations

Associations between iciHHV-6 status and baseline characteristics recorded on the GS:SFHS pre-clinical questionnaire and measurements collected at the research clinic visit were assessed using Fisher’s exact test for categorical variables and ANOVA for continuous traits. Associations between iciHHV-6 status and binary disease outcomes were evaluated using generalized linear mixed models, with the inclusion of a family identifier as a random effect to account for the relationships present within GS:SFHS (65, 66). Other variables added to adjust for known risk factors were included as fixed effects. All analyses compared iciHHV-6-positive with iciHHV-6-negative individuals, and then iciHHV-6A-positive and iciHHV-6B-positive individuals were compared with iciHHV-6-negative individuals. Analyses were performed using the statistical software platform R (67). Participants self-reporting ≥8 of the 15 medical conditions in the pre-clinical questionnaire were considered unreliable and excluded from these analyses (*n* = 25).

### Prediction of viral ancestral lineage

#### Strategy and nomenclature

A panel of assays, described below, was used to identify the ancestral lineage of iciHHV-6 genomes. Clade nomenclature is as described in Aswad et al. (2021) (41) with the addition of clade B9, which includes iciHHV-6B genomes integrated into chromosome 17p and is typified by the 1-ciHHV-6B cell line (3). Junction fragment sequences and viral lineages are named according to viral species, clade, and chromosome end, e.g., A2_17p, B8_17p, and B9_17p.

#### Clade-specific viral SNP genotyping assays

K-mer analysis was used to identify 41-mers unique to HHV-6B clades B4, B5, B6, and B8 for facilitating the identification of SNPs specific to these clades. Custom clade-specific viral SNP genotyping assays (Thermo Fisher Scientific) were designed to detect the B4-specific SNP at position 67,748, B5-specific SNP at position 47,851, B6-specific SNP at position 100,966, B8-specific SNP at position 60,158, and a C > T variant at position 42,889 in some clade B6 genomes (Table S3). All positions are relative to the HHV-6B reference sequence NC_000898.1. SNP genotyping assays were performed according to the manufacturer’s instructions (Thermo Fisher Scientific). DNA from the 2B-9q34.3, 4B-11p15.5, and COR264 cell lines was used as positive controls, and water was used as a no-template control. Thermal cycling and SNP detection were performed on a 7500 Fast Real-Time PCR system (Thermo Fisher Scientific). All GS:SFHS samples with available DNA were analyzed using the clade B6-specific assay. Samples negative for the B6 clade-specific SNP were then assayed using the B4, B5, and B8 assays, as appropriate. Selected samples with clade B6 genomes were tested for the B6-specific T variant at position 42,889.

#### HHV-6 whole-genome sequencing using targeted enrichment and Illumina methodology

HHV-6 genomes in 21 iciHHV-6-positive samples were sequenced using targeted enrichment and Illumina short-read sequencing as part of this study: 16 to verify clade and 1 to characterize a deletion mutant. DNA samples were sonicated using the Covaris LE220 to an approximate size of 450 bp. For library preparation, the Kapa LTP Library Preparation Kit for Illumina Platforms was used (Kapa Biosystems), as per the manufacturer’s instructions until adapter ligation. At this stage, samples were uniquely indexed using the NEBNext Multiplex Oligos for Illumina, 96 Unique Dual Index Primer Pairs Set 2 (New England Biolabs, Herts, UK) by using 12 PCR cycles. The amplified libraries were quantified using the Qubit dsDNA HS Kit (Thermo Fisher Scientific) and analyzed on an Agilent 4200 Tapestation System using High Sensitivity D5000 Screentape and High Sensitivity D5000 Reagents (Agilent Technologies Inc., CA, USA).

Hybridization and capture were performed using a SureSelectXT Custom 1–499 kb target enrichment system with probes designed for HHV-6 (Agilent Technologies Inc.) according to the manufacturer’s protocol. Sequencing of the libraries was carried out on an Illumina MiSeq System (Illumina, Cambridge, UK) using a MiSeq v3 600 cycle kit (Illumina). The pools were loaded at a final concentration of 12 pM using 2 × 251 read length and 2 × 8 bp index reads to produce approximately 50 million paired-end reads.

Reads with a quality score of <30 were discarded using TrimGalore v0.6.6 (https://github.com/FelixKrueger/TrimGalore). The remaining reads were aligned to U1102 (HHV-6A, accession: X83413.2) or HST (HHV-6B, accession: AB021506) using Burrows-Wheeler Aligner v0.7.17 (https://doi.org/10.48550/arXiv.1303.3997). Consensus sequences for each sample were generated using SAMtools v1.16.1 (68). Consensus sequences were aligned using MAFFT v7.475 (69), and the alignment was trimmed manually to remove the R1-3 repeats in the U region, DRs, and longer single nucleotide repeats. Maximum likelihood phylogenetic trees were inferred using the Tamura-Nei model (MEGA-X v10.2.5) (51).

Reads that mapped to the viral genome at the integration site were extracted using ReadMapper (https://github.com/deprekate/ReadMapper) using target sequences in DR. Reads extending into the telomere-repeat array in the integration site were processed using custom Python scripts and custom Microsoft Excel macros to convert telomere and telomere-like repeats into color-coded repeat maps (Fig. 3C).

#### Amplification and sequencing of viral T1 repeats

The proximal variable region of T1 in DR_R_ was amplified by nested PCR using DR1FRC paired with U100Fw2 in the primary reaction (94°C, 2 min; 25 cycles 94°C 15 s, 62°C 30 s, 68°C 10 min; 68°C 2 min) and DR421R paired with TJ1F in the secondary reaction (94°C, 2 min; 25 cycles 94°C 15 s, 64°C 30 s, 68°C 1.5 min; 68°C 2 min) with 1 µL 1:10 dilution of the primary PCR product (S3 Table). In samples with DR_1_-only iciHHV-6B genomes, this sequence was amplified without nested PCR using DR421R paired with TJ1F (94°C, 2 min; 35 cycles 94°C 15 s, 64°C 30 s, 68°C 1.5 min; 68°C 2 min; Table S3). PCR products were analyzed by agarose gel electrophoresis, and fragments were directly sequenced using Sanger sequencing (Source Bioscience) with primer TJ1F.

A DR_R_-T1-containing amplicon was PCR amplified from iciHHV-6A samples using primer A-T1seqDR paired with A-T1seqU (94°C, 2 min; 30 or 33 cycles 94°C 15 s, 57°C 30 s, 68°C 4 min; 68°C 2 min). PCR products were analyzed by agarose gel electrophoresis and grouped by fragment size.

#### Targeted locus amplification

Cells from the 2B-9q34.3, 4B-11p15.5, and 1-ciHHV-6B lines (51) were harvested 24 hours after subculturing and prepared for TLA using a Sample Cross-Link kit (Cergentis B.V., Utrecht, The Netherlands). Ten million cells were pelleted, washed, and incubated with fixation buffer FB (Cergentis B.V.) to cross-link DNA. Cross-linked DNA was subjected to enzymatic restriction digest with restriction buffer RB (Cergentis B.V.). Cells were resuspended in RB, snap frozen, and shipped to Cergentis B.V. for TLA analysis. Briefly, fragmented cross-linked DNA was religated, resulting in DNA fragments from the same spatial locus forming long, circular DNA strands. Circularized DNA was decross-linked, and outward-facing pairs of primers (DR1-FW and DR1 RV, and U99-FW and U99-RV) were used to amplify DNA circles. Amplified DNA was purified, and a sequencing library was prepared using the Nextera DNA Flex Library Prep protocol (Illumina) and Nextera DNA CD Indexes (Illumina). Paired-end sequencing (2 × 151 nt) was performed on a MiniSeq System (Illumina).

#### Optical genome mapping

Ultra-high molecular weight DNA was isolated from the 2B-9q34.3 cell line and cryopreserved whole blood samples from two selected iciHHV-6B-positive GS:SFHS participants using the SP Blood and Cell DNA Isolation Kit (Bionano Genomics, San Diego, USA). For each sample, approximately 750 ng of DNA was fluorescently labeled using the Direct Label and Stain kit (Bionano Genomics) following the Direct Label and Stain Kit Protocol (Bionano Genomics). DNA was fluorescently tagged using DLE-1 (Bionano Genomics) at CTTAAG recognition sites, and dsDNA was counterstained with DNA Stain (Bionano Genomics). Imaging was performed using the Bionano Genomics Saphyr System. Samples were loaded on a Saphyr Chip G2.3 (Bionano Genomics) and run on the Saphyr until 70× effective coverage of the human genome was obtained or for a maximum of 72 hours.

Analysis of read data from the instrument was carried out using Bionano Access version 1.7 (Bionano Genomics). Reads were assembled into consensus optical density maps (cmaps) using the *de novo* assembly option with the human optical density map (hg38 DLE-1) as a reference. The HHV-6 reference genome sequence was used to generate an *in silico* density map of the virus, which was used as part of the hybrid analysis. Where there was no loss of labels within the HHV-6 sequence, its alignment with contigs within the assembly generated from the optical density maps was readily detected, and those contigs linked to specific regions of the human genome. In samples where the HHV-6 sequence was not detected by hybrid analysis, a visual inspection of the telomeric region optical density maps was carried out.

#### Characterization of integration sites in chromosome 9q using Oxford Nanopore Technology whole-genome sequencing

DNA from the 2B-9q34.3 cell line was extracted using Genomic-tips (Qiagen, Manchester, UK). A sequencing library was prepared using the Ligation Sequencing Kit (SQK-LSK109) and sequenced for 20 hours on a MinION R9.4.1 flow cell (Oxford Nanopore Technologies, Oxford, UK) operated on a GridION Mk1 (Oxford Nanopore Technologies). DNA was extracted from whole blood samples from two GS:SFHS participants using the Monarch HMW DNA Extraction Kit for Cells and Blood (New England Biolabs) and sequenced for up to 72 hours on a MinION R10.4.1 flow cell following library preparation using the Ligation Sequencing Kit (SQK-LSK114).

Adapter sequences were removed from nanopore sequencing reads using Porechop v0.2.4 (https://github.com/rrwick/Porechop). Reads were aligned using Minimap2 (70) to subtelomere regions from the human reference genome (GRCh38) or to modified HHV-6A or HHV-6B reference genomes with an extended telomere repeat array at the predicted integration site.

#### Viral integration site assays

All iciHHV-6-positive samples were assayed using a chromosome 17p integration site assay, which amplifies A2_17p, B8_17p, and B9_17p junction fragments (2, 10, 34, 44, 71) (Table S3). iciHHV-6B-positive samples with clade B6 genomes were also assayed using two novel 9q integration site assays, which amplify two related B6_9q junction fragments, B6_9q_T2 and B6_9q_T1. Reactions contained 20–50 ng of template DNA, primers at 500 nM, 1× AccuPrime Buffer II, and 1 U of AccuPrime Taq High Fidelity enzyme (Thermo Fisher Scientific) in a final volume of 25 µL. Primer combinations were: St17p paired with HHV-6 DR_R_ for A2_17p and B8_17p; St17p paired with DR8F(A/B) for B9_17p; 9q_TJ1 paired with DR8F(A/B) for B6_9q_T2; and 9q_TJ1 paired with U100Fw2 for B6_9q_T1. DNA from the 7A-17p13.3 and 2B-9q34.3 cell lines was used as positive controls for the 17p and B6_9q_T2 integrations, respectively (3). GS:SFHS samples were used as positive controls for amplification of the B6_9q_T1 junction. Thermal cycling was performed on a Bio-Rad Laboratories C1000 Touch Thermo Cycler (94°C for 2 min; 31 or 40 cycles of 94°C for 15 s, 64°C for 30 s, 68°C for 2 min; 68°C 7 min [17p] or 2 min [9q]).

PCR products were analyzed by agarose gel electrophoresis. All B8_17p junction fragment amplicons, 19 B6_9q_T2 amplicons, and two B9_17p amplicons were sequenced on both strands using Sanger sequencing; dGTP chemistry was used for the G-rich strand (Source BioScience, Lanarkshire, UK). Sequencing reactions utilized primers St17p or 9q_TJ1 and either HHV-6A_ins_seq (A2_17p), HHV-6B_ins_seq3 (B8_17p), or DR8FT2 (B6_9q_T2 and B9_17p; Table S3). B6_9q junction fragments were scored largely on the basis of assay and amplicon size.

#### Genome-wide genotyping and identification of host haplotypes associated with iciHHV-6 genomes

Genome-wide genotyping of GS:SFHS samples was performed using the Illumina HumanOmniExpressExome-8 v1.0 BeadChip (first 9863 samples) and the Illumina HumanOmniExpressExome-8 v1.2 BeadChip, with Infinium chemistry. Data were available for 19,967 GS:SFHS participants with known iciHHV-6 status (Table S1).

The genotyped data were imputed using the Sanger Imputation Service (https://imputation.sanger.ac.uk/; accessed on 2 March 2017) using the Haplotype Reference Consortium panel v1.1 (http://www.haplotype-reference-consortium.org/particpating-cohorts) (72). Autosomal haplotypes were pre-phased using SHAPEIT2 v2r837 (73, 74), using the SHAPEIT2 duoHMM option11 (75), taking advantage of the cohort family structure to improve the imputation quality (76). Monogenic and low imputation quality (INFO < 0.4) variants were removed from the imputed dataset leaving 24,111,857 variants available for downstream analyses.

Family-based association analysis of the genotyped data was performed using the transmission disequilibrium test in PLINK (77). The association analysis with the Haplotype Reference Consortium imputed variants was performed with the software RegScan v0.2 (78). The pgresidualY estimated from the polygenic function in GenABEL was used for association analysis. The effect size, SEs, and *P* values were thereafter corrected to account for relatedness using the GRAMMAR-Gamma factors also provided by the polygenic function (79).

Genome-wide association analyses comparing iciHHV-6-positive participants with iciHHV-6-negative participants (controls) were performed separately for iciHHV-6A and iciHHV-6B. For iciHHV6B, the phased genotyping data were then used to identify host haplotypes at the ends of chromosomes 7p, 9q, 11p, 17p, and 19q that were associated with iciHHV-6B-positivity and in likely linkage disequilibrium with the integrated virus. Variants with an imputation probability ≥0.8 and *P* ≤ 0.01 for iciHHV-6B-association were used in the construction of haplotypes. To identify haplotypes in 9q, 11p, and 19q, families were identified in which the lead SNPs co-segregated with iciHHV-6B positivity. Data from PCR analysis of the 17p integration site were used to identify individuals with the B8_17p integration and the related haplotype. Individual participants with the lead 7p SNPs were used to identify the iciHHV-6B-associated haplotype. Following the identification of iciHHV-6-associated haplotypes in these selected GS:SFHS participants, the phased genotypic data from all participants were interrogated using VBA Macros in Microsoft Excel (Microsoft 365 MSO, Version 2303 Build 16.0.16227.20202) to identify the defined haplotypes.

Following the first round of haplotype assignment, the iciHHV-6B-positive individuals were divided into seven groups, and the genome-wide association analysis was repeated in an iterative fashion with subsequent refinement of the iciHHV-6B-associated haplotypes.

Provisional iciHHV-6A-associated host haplotypes were identified following the grouping of samples based on results from the chromosome 17p integration site assay, HHV-6 WGS, and DR_R_-T1 length. Variants with an imputation probability ≥0.8 were used in the construction of haplotypes.

For almost all samples, haplotype and clade assignments were concordant; therefore, clade names were added to the haplotype descriptors.

## Data Availability

All assembled sequences generated in this study have been deposited in GenBank under accession numbers PQ577348-PQ577367.

## SUPPLEMENTAL MATERIAL

Supplemental material jvi.02160-24-s0001.pdf Figures S1 to S5; Tables S1 to S10.

ASM does not own the copyrights to Supplemental Material that may be linked to, or accessed through, an article. The authors have granted ASM a non-exclusive, world-wide license to publish the Supplemental Material files. Please contact the corresponding author directly for reuse.
