# Supplementary material for: Inherited chromosomally integrated human herpesvirus 6: regional variation in prevalence, association with angina, and identification of ancestral viral lineages in two large UK studies
Source: J Virol. 2025 Jun 5;99(7):e02160-24. doi: 10.1128/jvi.02160-24 (PMC12282103; doi:10.1128/jvi.02160-24)
Supplement: Supplemental material — Figures S1 to S5; Tables S1 to S10. [file jvi.02160-24-s0001.pdf]

Supporting Information

for

Inherited chromosomally integrated human herpesvirus 6: regional variation in prevalence, association with angina, and identification of ancestral viral lineages in two large UK studies

Michael L Wood[1], Adam J Bell[1], Robin Young[2], Christopher Brownlie[1], Nick Orr[3], Archie Campbell[4,5,8], Jenna Nichols[1], Konstantinos Papageorgiou[1], Annette Lake[1], Nicolas M Suarez[1], Katherine Smollett[1], Natasha Jesudason[1], Salvatore Camiolo[1], Sreenu Vattipally[1], Joseph Hughes[1], Kirby Brown[1], Leah M Hunter[1], Euan Shaw[1], Skye Storrie[1], Rithu Paul Stansilaus[1], Eillis Sweeney[1], Tingyi Zhu[1], Angie Fawkes[6], Lee Murphy[6], William Tyne[7], Philip Howard[7], Michael E Jones[3], Katarzyna Tomczyk[3], Anne Richmond[4], James F Wilson[4,8], Duncan A Clark[9], Christian Delles[10], Nicola Royle[11], Shona M Kerr[4], Ana da Silva Filipe[1], Andrew Davison[1], Alex McConnachie[2], Anthony J Swerdlow[3], Caroline Hayward[4,5], Ruth F Jarrett[1].

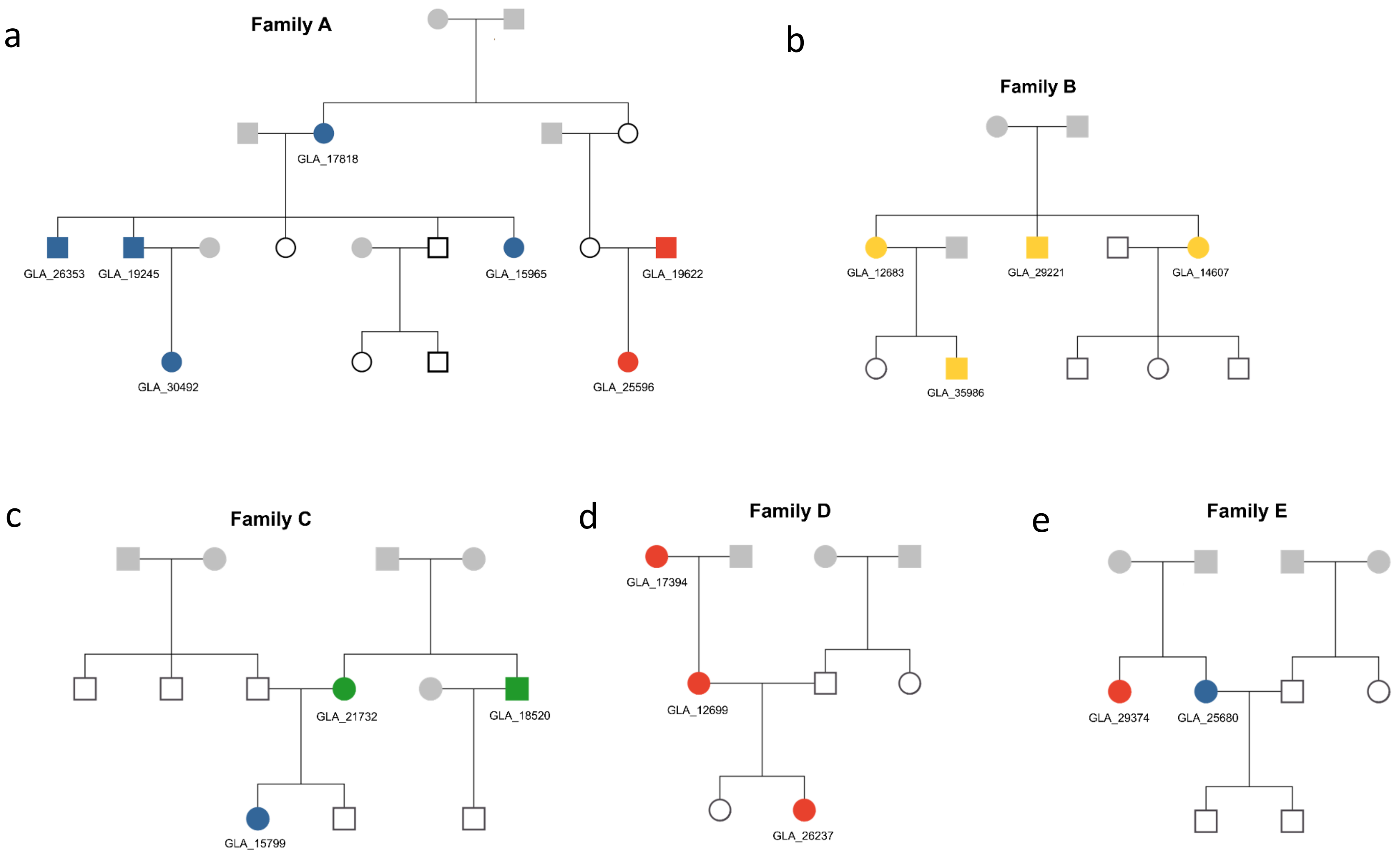

S1 Fig: iciHHV-6-positive family trees with unusual features

Squares, males; circles, females; grey, not tested; blue, complete genomes U_1DR_2; red, DR-only but in Family A denotes genomes with deletion of one direct repeat (DR) and all or most of the unique (U) region, $U_{partial}DR_1$; yellow, U_2DR_3; green, U_2DR_4. (a) Family A includes individuals with complete iciHHV-6B genomes and individuals with iciHHV-6A genomes with deletion of one DR and most of the U region. The viral genome in GLA_25596 was sequenced (Figure S5) and the genome in GLA_19622 was analysed using multiple PCR assays and provided consistent results regarding the location of the deletion. GLA_25596 is integrated in 17p. (b) Family B includes four iciHHV-6B-positive individuals with a U_2DR_3 composition suggesting that these genomes are inherited as a single genetic element. (c) Family C includes iciHHV-6B-positive individuals with genome compositions U_2DR_4 and U_1DR_2 consistent with independent inheritance of two viral genomes. (d) Inheritance of DR-only genomes. (e) Two siblings with different genome compositions - U_1DR_2 and DR-only – suggesting that deletion of U and one DR may have occurred in the germline of the affected parent or at fertilisation. Both siblings have the B6_9q_hap2 haplotype and B6_9q_T1 junction fragment.

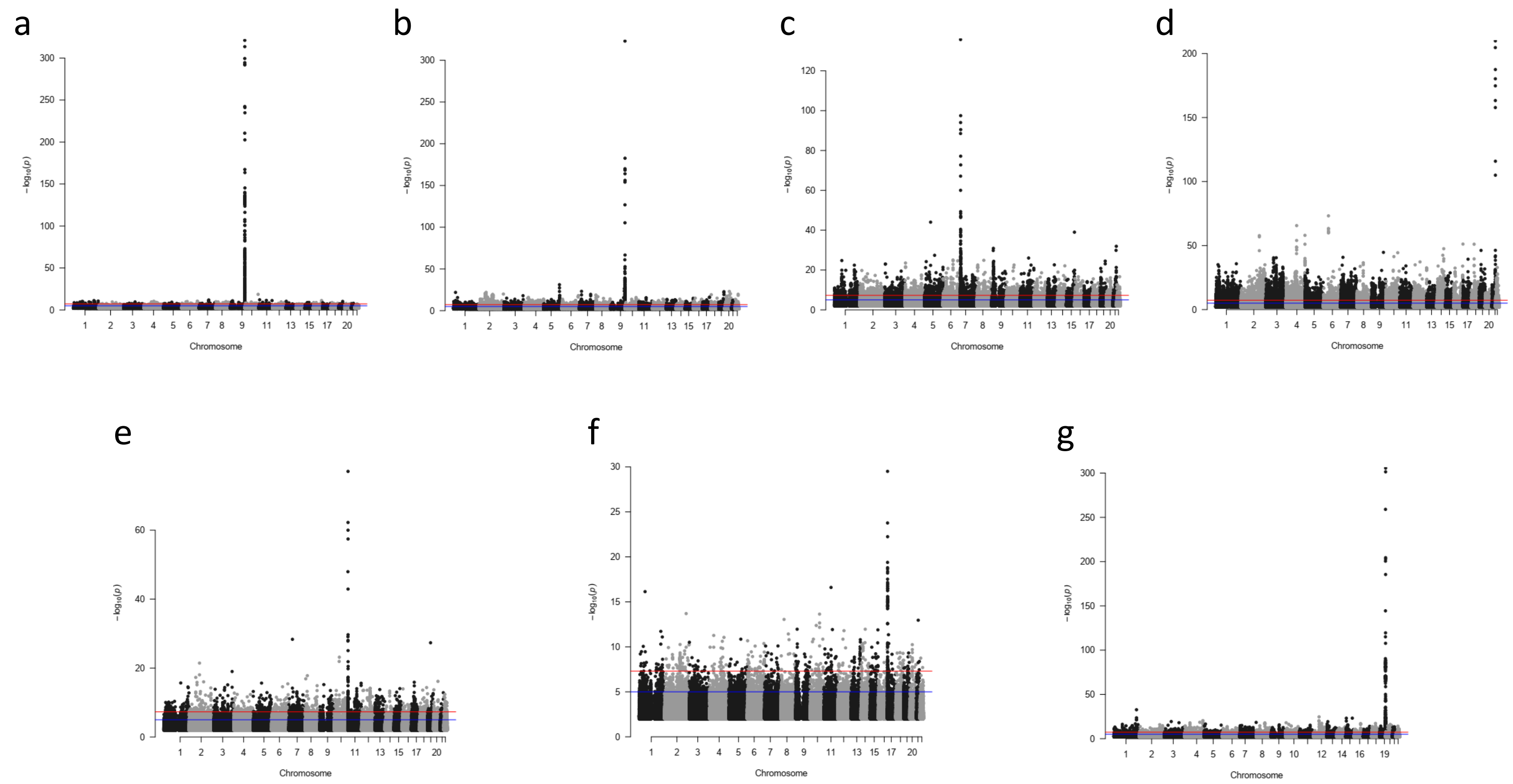

S2 Fig: Genome-wide association analysis of iciHHV-6B-positive individuals grouped by iciHHV-6B-associated haplotype

Following identification of iciHHV-6B-associated host haplotypes, and the first round of haplotype assignment, the iciHHV-6B-positive individuals were divided into seven groups based on haplotype and the presence of the clade B6-specific SNP (B6_7p; B6_9q_hap1; B6_9q_hap2; B4_11p; B8_17p; B5_19q; 'clade B6 genome but no identifiable haplotype'; and 'no identifiable haplotype'), and the genome-wide association analysis repeated. The Manhattan plot of the 'clade B6 genome but no identifiable haplotype' group showed peaks at 7p and 21q and a residual peak at 9q. This led to the identification of a 21q haplotype associated with B6 genomes, and a second version of the B6_7p haplotype, which diverged at rs139107235 (position 565202).

Removal of a small number of SNPs in B6_9q_hap1 led to loss of the residual signal at 9q in the ‘clade B6 but no identifiable haplotype’ group. The plot of the 'no identifiable haplotype' group initially showed a residual peak at 19q and led to shortening of the 19q haplotype. These changes resulted in more individuals being assigned to haplotype groups, and to loss of the signal in the Manhattan plots of the groups with no identifiable haplotype. In the final analysis shown here, the top row of Manhattan plots shows the analysis of the four haplotypes associated with clade B6 genomes: (a) B6_9q_hap1. (b) B6_9q_hap2. (c) B6_7p. (d) B6_21q. Bottom row: (e) B4_11p. (f) B8_17p. (g) B5_19q. The red and blue horizontal lines indicate *P* values of $5x10^{-8}$ and 10^{-5}, respectively. Note that the y axis scale showing the $-\log_{10}(P)$ varies in different plots.

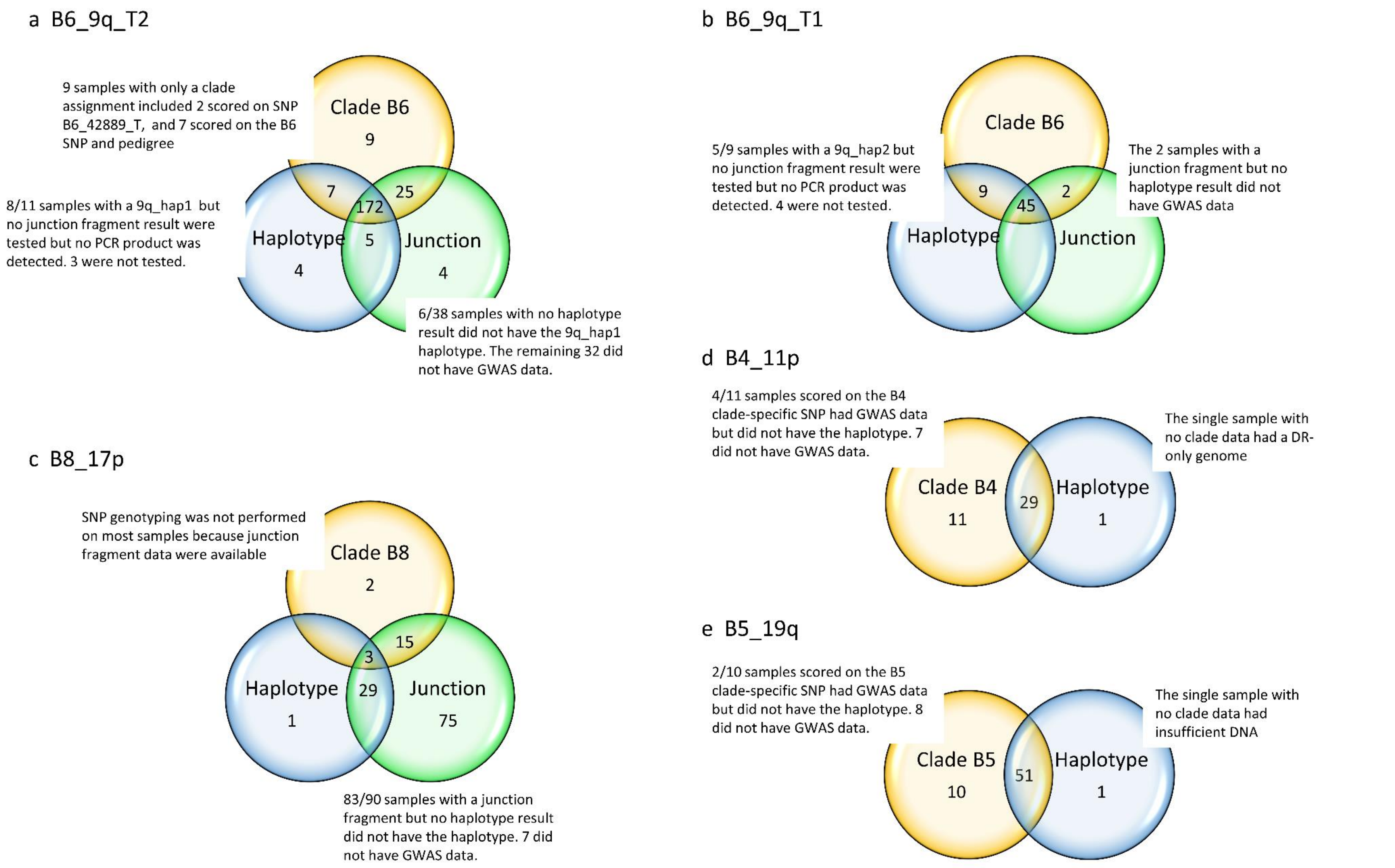
a B6_9q_T2
Clade B6
9
9 samples with only a clade assignment included 2 scored on SNP B6_42889_T, and 7 scored on the B6 SNP and pedigree
7
25
172
5
Haplotype
4
Junction
4
8/11 samples with a 9q_hap1 but no junction fragment result were tested but no PCR product was detected. 3 were not tested.
6/38 samples with no haplotype result did not have the 9q_hap1 haplotype. The remaining 32 did not have GWAS data.
b B6_9q_T1
Clade B6
5/9 samples with a 9q_hap2 but no junction fragment result were tested but no PCR product was detected. 4 were not tested.
The 2 samples with a junction fragment but no haplotype result did not have GWAS data
9
45
2
Haplotype
Junction
c B8_17p
SNP genotyping was not performed on most samples because junction fragment data were available
Clade B8
2
15
3
Haplotype
1
29
Junction
75
83/90 samples with a junction fragment but no haplotype result did not have the haplotype. 7 did not have GWAS data.
d B4_11p
4/11 samples scored on the B4 clade-specific SNP had GWAS data but did not have the haplotype. 7 did not have GWAS data.
The single sample with no clade data had a DR-only genome
Clade B4
11
29
Haplotype
1
e B5_19q
2/10 samples scored on the B5 clade-specific SNP had GWAS data but did not have the haplotype. 8 did not have GWAS data.
The single sample with no clade data had insufficient DNA
Clade B5
10
51
Haplotype
1

S3 Fig: Concordance of assay results used to assign ancestral viral lineage of iciHHV-6B genomes

Venn diagrams showing the overlap of results from clade assignment by viral SNP genotyping or sequencing, host haplotype analysis, and virus:host junction fragment analysis. Samples outside the area of maximum intersection had missing data unless otherwise indicated. Missing data resulted from lack of GWAS data and, therefore, no haplotype information (n=70) and from DR_1-only genomes (n=20), which could not be SNP genotyped. DNA was also limiting in some instances. With the exception of the B8_17p lineage, the vast majority of results were concordant; lack of concordance was most frequently due to failure to detect a lineage-specific haplotype. Integration site assays generating junction fragments were available for only B6_9q, B8_17p, and B9_17p lineages.

(a) B6_9q lineage with the B6_9q_T2 junction fragment: 226 individuals. In this group, 172 individuals had the B_9q_hap1 haplotype, clade B6-specific SNP and B6_9q_T2 junction fragment; a further five had both the haplotype and the junction fragment. Eight of the thirteen individuals with no viral SNP genotyping results had DR_1-only genomes. Most (6/38) individuals who lacked haplotype information did not have GWAS data; however, there were 6 individuals who had the B6_9q_T2 junction fragment but did not have the B_9q_hap1 haplotype. There were also eight individuals with the B_9q_hap1 haplotype and no amplifiable B6_9q_T2 junction fragment, likely due to DNA quality and amount. There were nine individuals with only clade data; since we identified three viral lineages within clade B6, the B6 clade data alone cannot be used to assign lineage. Pedigree analysis was used to assign lineage for seven individuals in this group. The remaining two individuals had the T SNP at position 42889 of the HHV-6B reference genome, which we have found in only the B6_9q_T2 lineage.

(b) B6_9q lineage with the B6_9q_T1 junction fragment: 56 individuals. Classification in this group was dependent on having either the B6_9q_hap2 haplotype or the B6_9q_T1 junction fragment since the B6 SNP is not specific to this group. All tested samples had the C SNP at position 42889 of the HHV-6B reference genome. There were five samples from individuals with the B6_9q_hap2 haplotype that did not have an amplifiable B6_9q_T1 junction fragment; however, this junction fragment amplicon is 4.6 kb and it is possible that amplification failure was due to DNA quality.

(c) B8_17p lineage: 125 individuals. Classification in this lineage largely relied on amplification and sequencing of the B8_17p junction fragment. Of the 125 individuals in this group, 122 had the B8_17p_int1 junction fragment. Only 33 of the 118 individuals with GWAS data had the identified 17p-associated haplotype, including one who did not have a junction fragment result. A further two individuals with no junction fragment result were grouped in clade B8, based on presence of the B8-specific viral SNP (n=1) or sequence analysis of the proximal variable region of T1 (n=1). B8-specific genotyping was not routinely performed in individuals with a B8_17p junction fragment.

(d) B4_11p lineage: 41 individuals. There were 29 individuals with both the clade B4-specific SNP and the B_11p haplotype. A further individual with a DR_1-only genome was classified based on haplotype. Classification of 11 samples was based on detection of the clade B4-specific SNP. These included seven with no GWAS data, but there were four individuals with the clade-specific SNP but no detectable haplotype.

(e) B5_19q lineage: 62 individuals. There were 51 individuals in this group with both the B_19q_hap1 haplotype and the B5-specific SNP. Ten genomes were classified in this lineage based on the B5-specific SNP, including eight who did not have GWAS data. There were two individuals with the B5-specific SNP who did not have the associated haplotype.

Other lineages: Venn diagrams for the B6_7p (n=19) and B6_21q (n=11) lineages are not shown because classification in these lineages was dependent on having the associated haplotype. A small number of genomes were classified in these lineages based on possession of the clade B6-associated SNP and pedigree analysis, including three genomes in the B6_7p lineage, and one in B6_21q. Five genomes from two families were classified in the B9_17p lineage; all had the virus:host junction fragment that is specific for this lineage.

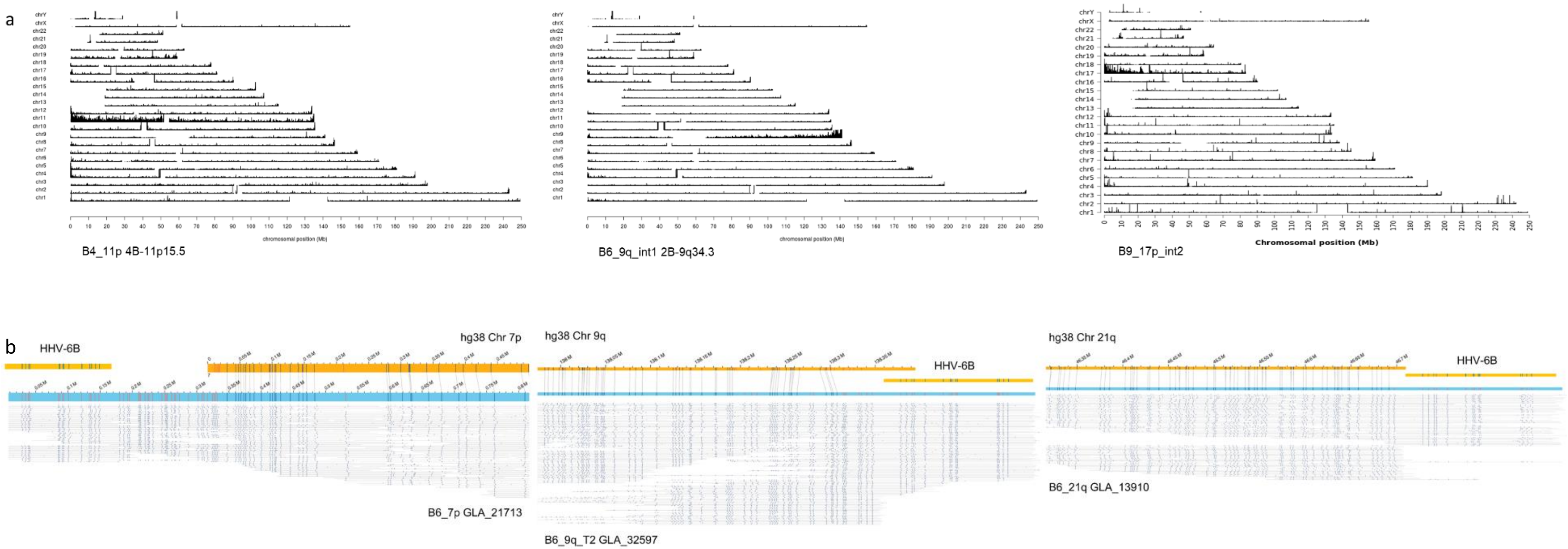

S4 Fig: Chromosomal location of iciHHV-6B determined by Targeted Locus Amplification and Optical Genome Mapping

(a) Whole genome coverage plots generated from short-read sequence data from Targeted Locus Amplification (TLA) carried out on three different iciHHV-6B positive cell lines, 4B-11p15.5, 2B-9q34.3 and 1-ciHHV-6B with predicted viral integration sites at 11p, 9q and 17p, respectively. Read depth was highest in the 11p, 9q and 17p arms of these samples, respectively, confirming the predicted integration sites.

(b) Consensus maps (cmaps, light blue) from Optical Genome Mapping (OGM). Overlapping optical maps of DNA molecules fluorescently labelled at CTTAAG sequence motifs from three iciHHV-6B positive samples (GLA_21713, GLA_26419, and GLA13910) were aligned to the human reference (hg38). The HHV-6-specific fluorescent label pattern signal was identified at the end of cmaps that aligned to 7p, 9q and 21q for samples GLA_21713, GLA_32597, and GLA_13910, respectively. As a result of population-level variation in the subtelomere region not represented in the human reference genome, cmaps at the end of chromosome arms do not necessarily map precisely to the reference.

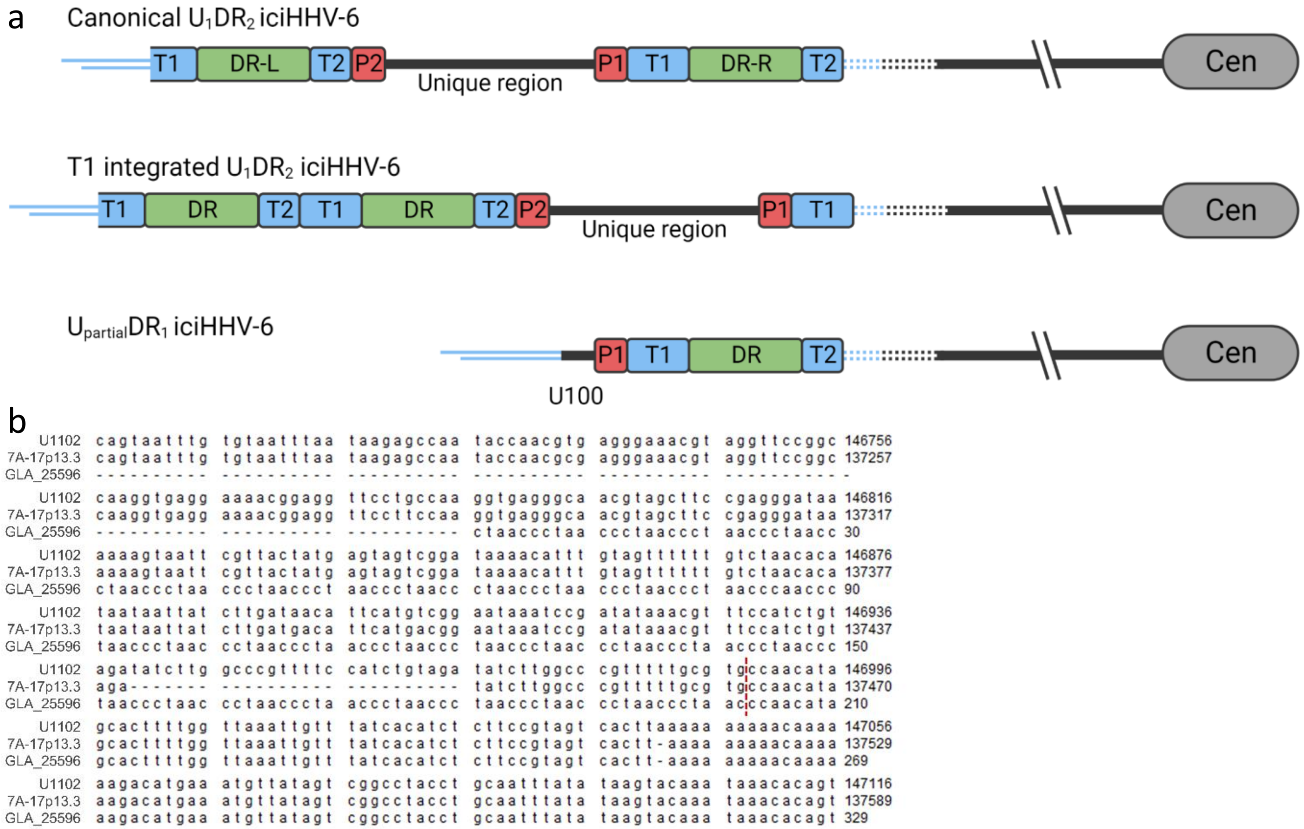

S5 Fig: Viral genome structure of the B6_9q_T1 and A2_17p_$U_{partial}DR_1$ viruses

(a) Comparison between the canonical U_1DR_2 genome structure integrated at DR_R-T2 (top) and non-canonical iciHHV-6 genomes. The middle panel shows the rearranged U_1DR_2 genome, observed in the B6_9q lineage, with concatemeric DR regions in a telomeric position and the viral DR_R-T1 at the integration site (B6_9q_T1). The bottom panel shows the non-canonical genome of some A2_17p lineage iciHHV-6A genomes that retain a single DR, including DR-T1, and several hundred bases of the unique region ($U_{partial}DR_1$) following the loss of the second DR and most of the unique region.

(b) Sequence alignment of part of U100 from an exogenous HHV-6A genome (U1102, GenBank X83413.2), a canonical A2_17p U_1DR_2 genome (7A-17p13.3, GenBank KY316048), and a non-canonical A2_17p_$U_{partial}DR_1$ genome (GLA_25596). The breakpoint in the $U_{partial}DR_1$ genome is at position 146988 in the HHV-6A reference genome U1102 indicated by the dashed red line. There is no evidence of telomere repeats in the HHV-6A genome at this position, but short-read sequencing reveals the presence of a telomere-repeat array 5' to the breakpoint that is at least 200 bp in length.

S1 Table: Characteristics of GS:SFHS participants

Demographic	Initial screen	Successful iciHHV-6 screen	GWAS available*
	**Number (%)**	**Number (%)**	**Number (%)**
**Participants**	23930 (100)	23637 (100)	19967 (100)
**Families**	6930 (100)	6913 (100)	6650 (100)
**Sex**			
Female	14062 (58.8)	13894 (58.8)	11758 (58.9)
Male	9868 (41.2)	9743 (41.2)	8209 (41.1)
**Age Group (years)**			
18-24	2160 (9.0)	2130 (9.0)	1803 (9.0)
25-34	3246 (13.6)	3204 (13.6)	2609 (13.1)
35-44	4389 (18.3)	4341 (18.4)	3715 (18.6)
45-54	5175 (21.6)	5122 (21.7)	4477 (22.4)
55-64	6037 (25.2)	5973 (25.3)	5196 (26.0)
65-74	2104 (8.8)	2075 (8.8)	1651 (8.3)
≥75	816 (3.4)	792 (3.4)	516 (2.6)
**Nationality**			
Scottish	20021 (83.66)	19807 (83.80)	17396 (87.1)
English	1571 (6.56)	1527 (6.46)	877 (4.39)
Northern Irish	55 (0.23)	54 (0.23)	33 (0.17)
Welsh	51 (0.21)	46 (0.19)	25 (0.13)
Irish†	235 (0.98)	230 (0.97)	174 (0.87)
Pakistani	23 (0.1)	23 (0.10)	-
Indian	45 (0.19)	45 (0.19)	-
Chinese	12 (0.05)	12 (0.05)	-
African	9 (0.04)	9 (0.04)	-
Caribbean	9 (0.04)	9 (0.04)	-
Other	408 (1.70)	405 (1.71)	309 (1.55)
Missing	1491 (6.23)	1470 (6.22)	1153 (5.77)
**Ethnicity**			
White	22685 (94.80)	22406 (94.79)	19202 (96.2)
Black	16 (0.07)	16 (0.07)	-
Asian	112 (0.47)	112 (0.47)	-
Mixed	108 (0.45)	108 (0.46)	4 (0.02)
Other	24 (0.10)	24 (0.10)	2 (0.01)
Missing	985 (4.12)	971 (4.10)	759 (3.80)

Sample screened			
**DNA from blood**	21387 (89.4)	21256 (90)	19391 (97.1)
**DNA from saliva**	2543 (10.6)	2381 (10)	576 (2.9)

*Includes only those with successful screening result; †participants who self-identified as being from the Republic of Ireland

S2 Table: Characteristics of BGS participants and iciHHV-6 positivity by case control status, country and region

	Total	iciHHV-6+ all	iciHHV-6A+	iciHHV-6B+
		Number (%)	Number (%)	Number (%)
**All**	7910	90 (1.14)	20 (0.25)	70 (0.88)
**Control***	3943	43 (1.09)	10 (0.25)	33 (0.83)
**Case***	3967	47 (1.18)	10 (0.25)	37 (0.93)
Incident case	1316	16 (1.22)	4 (0.30)	12 (0.92)
Prevalent case	2651	31 (1.17)	6 (0.23)	25 (0.94)
**Age (years)#**				
16 - 24	46	1 (2.2)	0 (0)	1 (2.2)
25 - 34	418	12 (2.9)	3 (0.7)	9 (2.2)
35 - 44	1737	18 (1.0)	2 (0.1)	16 (0.9)
45 - 54	2990	28 (0.9)	9 (0.3)	19 (0.6)
55 - 64	2124	23 (1.1)	4 (0.2)	19 (0.9)
65 - 74	540	6 (1.1)	2 (0.4)	4 (0.7)
≥75	55	2 (3.6)	0 (0)	2 (3.6)
**Country of residence**				
**England**				
All	7111	78 (1.10)	17 (0.24)	61 (0.86)
Control	3544	37 (1.04)	8 (0.23)	29 (0.82)
Case	3567	41 (1.15)	9 (0.25)	32 (0.90)
**Scotland**				
All	453	8 (1.77)	1 (0.22)	7 (1.55)
Control	230	4 (1.74)	1 (0.43)	3 (1.30)
Case	223	4 (1.79)	0 (0)	4 (1.79)
**Northern Ireland**				
All	42	0 (0)	0 (0)	0 (0)
Control	19	0 (0)	0 (0)	0 (0)
Case	23	0 (0)	0 (0)	0 (0)
**Wales**				
All	304	4 (1.32)	2 (0.66)	2 (0.66)
Control	150	2 (1.33)	1 (0.67)	1 (0.67)
Case	154	2 (1.30)	1 (0.65)	1 (0.65)
**Regions of England**				
**North of England**				

All	1453	22 (1.51)	4 (0.28)	18 (1.24)
Control	723	12 (1.66)	2 (0.28)	10 (1.38)
Case	730	10 (1.37)	2 (0.27)	8 (1.10)
**Central England**				
All	1313	14 (1.07)	1 (0.08)	13 (0.99)
Control	653	10 (1.53)	1 (0.15)	9 (1.38)
Case	660	4 (0.61)	0 (0)	4 (0.61)
**South of England**				
All	3783	42 (1.11)	12 (0.32)	30 (0.79)
Control	1881	15 (0.80)	5 (0.27)	10 (0.53)
Case	1902	27 (1.42)	7 (0.37)	20 (1.05)
**East Anglia**				
All	562	0 (0)	0 (0)	0 (0)
Control	287	0 (0)	0 (0)	0 (0)
Case	275	0 (0)	0 (0)	0 (0)

*Cases are BGS participants with breast cancer, controls are participants without breast cancer; incident case, diagnosed with breast cancer after study entry; prevalent case, diagnosed with breast cancer before study entry; #age at blood draw.

S3 Table: Primers and probes

Primer/Probe Name	Reference	Sequence and probe fluorochromes
B-globin Forward	Saiki et al, 1988	GGCAACCCTAAGGTGAAGGC
B-globin Reverse	Saiki et al, 1988	GGTGAGCCAGGCCATCACTA
B-globin Probe	Saiki et al, 1988	6-FAM-ATGGCAAGAAAGTGCTCGGTGCCT-TAMRA
HHV-6 DR1 Forward		GAAACTGTAACGGCCACGTT
HHV-6 DR1 Reverse		GTGCTCCG**C**CACGACTAC
HHV-6 DR1 Probe		HEX-CGCCGCCGCCGTTACTGTC-BHQ1
HHV-6 DR6A Forward		CGGCATCGCGGAGAAC
HHV-6 DR6A Reverse		TGTACGGATCGTGGTGGAGTT
HHV-6 DR6A Probe		HEX-CCACCTACCGTGGCCGCCGT-BHQ1
HHV-6 DR6B Forward		GCAGGCCGTCCAAACTGT
HHV-6 DR6B Reverse		ACGGTAGGTGGATCCGTTCTC
HHV-6 DR6B Probe		HEX-CGGCTATACGAGTCGGCACCGG-BHQ1
HHV-6 U7 Forward	Tavakoli et al, 2007	AAAATTTCTCACGCCGGTATTC
HHV-6 U7 Reverse	Tavakoli et al, 2007	CCTGCAGACCGTTCGTCAA
HHV-6 U7 Probe	Tavakoli et al, 2007	6-FAM-TCGGTCGACTGCCCGCTACCA-TAMRA
HHV-6 POLA Forward	Gallagher et al, 2002	GGATGAGAC**T**CATCGGTTTGTG
HHV-6 POLA Reverse	Gallagher et al, 2002	GGCCAGCCAGTCCTTTAGTAGA
HHV-6 POLA Probe	Gallagher et al, 2002	HEX-TCCCAAGCACAGACTC**A**CG**G**A**T**ACAAGG-BHQ1
HHV-6 POLB Forward	Gallagher et al, 2002	GGATGAGACCCATCGGTTTGTG
HHV-6 POLB Reverse	Gallagher et al, 2002	GGCCAGCCAGTCCTTTAGTAGA
HHV-6 POLB Probe	Gallagher et al, 2002	HEX-TTCCAAGCACAGACTC**G**CG**A**A**C**ACAAGG-BHQ1
HHV-6 U100A Forward		GGCCGTCGCCATCGA
HHV-6 U100A Reverse		AGGCTGAGCGCTATGAAACC
HHV-6 U100A Probe		FAM-AACGCGCACAAAAATATCAGCGCAC-3BHQ1
HHV-6 U100B Forward		GGTGGACCGTAGCATTCGA
HHV-6 U100B Reverse		GACGTTCTGCGCCGATACTC
HHV-6 U100B Probe		FAM-CTGTCGCCA-ZEN-TGGAAATCG-3IABkFQ
HHV-6 DR_R	Arbuckle et al, 2010	CATAGATCGGGACTGCTTGAAAGCGC
HHV-6 DR8F(A/B)	Wood et al, 2021	CATAGATCGGGACTGCTTGAA
St17p	Baird et al, 2003	AACATCGAATCCACGGATTGCTTTGTGTAC
9q_TJ1		CTTCTCAGCACAGACGTTGG
DR8FT2	Wood et al 2021	TCGGACCCTTGCTATTCTGG
DR1FRC	Wood et al 2021	GCATTCTTGCTCGGGCCAAGGT

U100Fw2	Wood et al 2021	TATCTCCGAACATGATGCTG
TJ1F	Wood et al 2021	AACCCTAAGTCTAGCCCTTG
DR421R	Wood et al 2021	GAGKGGTTGAAAGAGGGGTAG
A-T1seqDR		GGAAGGTGGTGATGGTGTGA
A-T1seqU		TGGTGGTCAGATATTAAGTC
**Clade-specific assays**	**SNP position**	
B4 Forward		ACGGCAAGATGACTAGACAGATGTA
B4 Reverse		GGCCAATCATAAGAAACTCAGAGACA
B4 Clade-specific probe		VIC-ACTTTAAATTCGT**A**AGCTGC
B4 Reference probe	67748	FAM-CTTTAAATTCGT**G**AGCTGC
B5 Forward		CGAGACAAGAATATTTTCGCACAACT
B5 Reverse		TGCACGTTTTCTGATTCGATAGGAA
B5 Clade-specific probe		FAM-TTTCGCC**A**AGTTTG
B5 Reference probe	47851	VIC-AGTTTCGCC**G**AGTTTG
B6 Forward		CATATGCATCAGACCTTCGTCACA
B6 Reverse		CCTGTGAACTTAGTTGATAGAAATCAATACC
B6 Clade-specific probe		VIC-CCGTCG**A**AAGTATC
B6 Reference probe	100966	FAM-CGTCG**G**AAGTATC
B6_42889 Forward		CCCATACTGGAGCTTTGATAAAACCT
B6_42889 Reverse		CCGCGGCAGCATTCC
B6_42889_C Probe	42889	VIC-ACCCAGTAC**G**TTTACG
B6_42889_T Probe	42889	FAM-CCCAGTACATTTACG
B8 Forward		CCCAATACGTTGAGATACATAAAAATTTCCAAAA
B8 Reverse		CGGCTGATAAGTTGTCATTGTATGG
B8 Clade-specific probe		VIC-CAACAGTGTTG**T**CATTGTA
B8 Reference probe	60158	FAM-CAGTGTTG**G**CATTGTA

HHV-6A and 6B POL primers/probe are highly similar; differences are indicated in bold font. Sequence analysis performed during this project showed that some iciHHV-6A genomes have a C>T change in the DR1 reverse primer sequence and a T>C change in the HHV-6A POL probe; both nucleotides are highlighted in red. Single nucleotide variants in the clade-specific assays are also shown in red (clade) and blue (reference); positions are relative to the HHV-6B reference sequence NC_000898.1.

S4 Table: Genome copy number and composition in iciHHV-6-positive study participants

	GS:SFHS				BGS	
	iciHHV-6A+		iciHHV-6B+		iciHHV-6A+	iciHHV-6B+
	Participant	Family	Participant	Family	Participant	Participant
**Genome composition**	**Number (%)**	**Number (%)**	**Number (%)**	**Number (%)**	**Number (%)**	**Number (%)**
U_1DR_2	34 (77.3%)	17 (68.0%)	567 (94.0%)	327 (93.2%)	13 (65%)	62 (89%)
U_2DR_4			3 (0.5%)	2 (0.6%)		1 (1.4%)
DR-only	1 (2.3%)	1 (4.0%)	20 (3.3%)	12 (3.4%)		4 (6%)
$U_{partial}DR_1$	2 (4.5%)	1 (4.0%)			1 (5%)	
U_2DR_3			8 (1.3%)	5 (1.4%)		1 (1.4%)
U_1DR_3	4 (9.1%)	4 (16.0%)	1 (0.2%)	1 (0.3%)	5 (25%)	
DR_2-only	2 (4.5%)	1 (4.0%)				
U_1DR_4	1 (2.3%)	1 (4.0%)				
U_1DR_1						1 (1.4%)
Not resolved			4 (0.66%)	4 (1.1%)	1 (5%)	1 (1.4%)
**TOTAL**	44	25*	603	351*	20	70

Genome compositions were assigned based on the mean copies per cell (mcpc) in the U7, DR6A and DR6B, and U100A and U100B assays. Samples categorised as DR_1-only or DR_2-only had one or two mcpc of DR6 and were negative in U7 and U100 ddPCR assays and the POL assays. Samples categorised as $U_{partial}DR_1$ had a single copy of DR6A and U100A but were negative in U7 and POL assays. Genome compositions in six iciHHV-6+ participants were not resolved because of insufficient DNA.

The 647 iciHHV-6+ participants in GS:SFHS were from 368 pedigrees. *The sum of iciHHV-6A+ and iciHHV-6B+ families in the 'Total' row exceeds the actual number of iciHHV-6+ families in the study because some families are represented more than once in the table columns. Two families included iciHHV-6A+ and iciHHV-6B+ participants and, in one of these families, the HHV-6B genomes were complete while the HHV-6A genomes had a single DR with partial deletion of U (Figure S5). Three families had a member with an unresolved genome composition as well as a member(s) with defined composition(s); two families had members with U_2DR_4 and U_1DR_2; and one family included siblings with different compositions, U_1DR_2 and DR_1-only (Figure S4). In all other families the genome compositions were consistent in affected family members and U_2DR_3, DR_1-only, DR_2-only, and $U_{partial}DR_1$ genomes were inherited within families (Figures S1).

S5 Table: iciHHV-6 status by birthplace of participant and parents, and by participant nationality, in GS:SFHS

Birthplace of participant	All Number	iciHHV-6+ Number (%)	iciHHV-6A+ Number (%)	iciHHV-6B+ Number (%)
Scotland	19496	555 (2.85)	34 (0.17)	521 (2.67)
England	1915	34 (1.78)	6 (0.31)	28 (1.46)
P value		0.0053	0.29	0.0018
**Birthplace of parents**				
Scotland/Scotland	16425	476 (2.9)	19 (0.21)	457 (2.78)
Scotland/England	1737	47 (2.71)	12 (0.69)	35 (2.01)
England/England	1172	16 (1.37)	7 (0.6)	9 (0.77)
P value		0.0065	0.0005	0.0005
**Nationality**				
Scottish	19807	577 (2.91)	35 (0.18)	542 (2.74)
English	1527	19 (1.24)	6 (0.39)	13 (0.85)
Northern Irish	54	2 (3.70)	0 (0)	2 (3.70)
Irish*	230	6 (2.61)	0 (0)	6 (2.61)
Welsh	46	0 (0)	0 (0)	0 (0)
Pakistani	23	0 (0)	0 (0)	0 (0)
Indian	45	0 (0)	0 (0)	0 (0)
Chinese	12	0 (0)	0 (0)	0 (0)
African	9	0 (0)	0 (0)	0 (0)
Caribbean	9	0 (0)	0 (0)	0 (0)
Other	833	12 (0.99)	0 (0)	12 (0.99)
Missing	1470	39 (2.66)	3 (0.20)	36 (2.45)
P value overall†		0.0025	0.52	0.0015
P value Scottish versus English†		<0.0001	0.070	<0.0001

*Participants who self-identified as being from the Republic of Ireland; †, Fisher's exact test.

S6 Table: Association between iciHHV-6 and laboratory measurements and selected characteristics in GS:SFHS

Variable	Statistic	All (N = 23637)	iciHHV-6– (N = 22990)	iciHHV-6+ (N = 647)	iciHHV-6A+ (N = 44)	iciHHV-6B+ (N = 603)
**Avg. Diastolic BP (mmHg)**	N_{obs} (N_{miss})	21277 (2360)	20686 (2304)	591 (56)	40 (4)	551 (52)
	Mean (SD)	79.5 (10.4)	79.5 (10.3)	79.9 (10.6)	81.2 (10.3)	79.8 (10.7)
	P value			0.34	0.30	0.47
**Avg. Systolic BP (mmHg)**	N_{obs} (N_{miss})	21277 (2360)	20686 (2304)	591 (56)	40 (4)	551 (52)
	Mean (SD)	131.2 (17.8)	131.2 (17.8)	130.9 (18.2)	133.3 (17.8)	130.7 (18.3)
	P value			0.65	0.46	0.50
**Avg. Heart Rate (bpm)**	N_{obs} (N_{miss})	21270 (2367)	20679 (2311)	591 (56)	40 (4)	551 (52)
	Mean (SD)	69.6 (11.3)	69.5 (11.3)	70.3 (10.8)	72.9 (9.9)	70.1 (10.9)
	P value			0.10	0.06	0.23
**Glucose (mmol/l)**	N_{obs} (N_{miss})	20038 (3599)	19483 (3507)	555 (92)	38 (6)	517 (86)
	Mean (SD)	4.8 (1.1)	4.8 (1.1)	4.8 (0.7)	4.6 (0.4)	4.8 (0.7)
	P value			0.41	0.19	0.62
**HDL Cholesterol (mmol/l)**	N_{obs} (N_{miss})	20247 (3390)	19687 (3303)	560 (87)	38 (6)	522 (81)
	Mean (SD)	1.5 (0.4)	1.5 (0.4)	1.5 (0.4)	1.6 (0.5)	1.4 (0.4)
	P value			0.90	**0.01**	0.41
**Total Cholesterol (mmol/l)**	N_{obs} (N_{miss})	20287 (3350)	19724 (3266)	563 (84)	38 (6)	525 (78)
	Mean (SD)	5.1 (1.1)	5.1 (1.1)	5.0 (1.1)	5.0 (1.0)	5.0 (1.1)
	P value			0.14	0.60	0.16
**Sodium (mmol/l)**	N_{obs} (N_{miss})	20309 (3328)	19746 (3244)	563 (84)	38 (6)	525 (78)
	Mean (SD)	139.9 (2.5)	139.9 (2.5)	139.8 (2.5)	140.5 (2.5)	139.8 (2.4)
	P value			0.44	0.14	0.23
**Potassium (mmol/l)**	N_{obs} (N_{miss})	20026 (3611)	19471 (3519)	555 (92)	37 (7)	518 (85)
	Mean (SD)	4.2 (0.4)	4.2 (0.4)	4.2 (0.4)	4.1 (0.3)	4.2 (0.4)
	P value			0.11	0.17	0.19
**Urea (mmol/l)**	N_{obs} (N_{miss})	20324 (3313)	19761 (3229)	563 (84)	38 (6)	525 (78)
	Mean (SD)	5.1 (1.5)	5.1 (1.5)	5.1 (1.4)	5.0 (1.5)	5.1 (1.4)
	P value			0.23	0.58	0.27
**Creatinine (mg/dl)**	N_{obs} (N_{miss})	20325 (3312)	19762 (3228)	563 (84)	38 (6)	525 (78)
	Mean (SD)	0.8 (0.2)	0.8 (0.2)	0.8 (0.3)	0.8 (0.1)	0.8 (0.3)
	P value			0.84	0.52	0.71

**Height (cm)**	N_{obs} (N_{miss})	21238 (2399)	20649 (2341)	589 (58)	40 (4)	549 (54)
	Mean (SD)	168.1 (9.5)	168.1 (9.5)	167.7 (9.7)	167.3 (7.2)	167.7 (9.8)
	P value			0.32	0.60	0.37
**Height (cm), Male**	N_{obs} (N_{miss})	8724 (1019)	8476 (995)	248 (24)	14 (1)	234 (23)
	Mean (SD)	176.2 (7.0)	176.2 (7.0)	176.0 (6.7)	173.9 (4.5)	176.1 (6.8)
	P value			0.75	0.22	0.98
**Height (cm), Female**	N_{obs} (N_{miss})	12514 (1380)	12173 (1346)	341 (34)	26 (3)	315 (31)
	Mean (SD)	162.5 (6.6)	162.5 (6.6)	161.7 (6.5)	163.8 (5.8)	161.5 (6.5)
	P value			**0.02**	0.32	**0.007**
**Weight (kg)**	N_{obs} (N_{miss})	21166 (2471)	20580 (2410)	586 (61)	40 (4)	546 (57)
	Mean (SD)	75.6 (16.5)	75.6 (16.5)	74.7 (15.9)	73.4 (12.5)	74.8 (16.2)
	P value			0.18	0.40	0.24
**Weight (kg), Male**	N_{obs} (N_{miss})	8707 (1036)	8460 (1011)	247 (25)	14 (1)	233 (24)
	Mean (SD)	83.5 (14.9)	83.6 (15.0)	82.7 (13.9)	77.3 (11.1)	83.0 (14.0)
	P value			0.36	0.12	0.58
**Weight (kg), Female**	N_{obs} (N_{miss})	12459 (1435)	12120 (1399)	339 (36)	26 (3)	313 (33)
	Mean (SD)	70.0 (15.2)	70.0 (15.2)	68.8 (14.7)	71.3 (12.9)	68.6 (14.9)
	P value			0.14	0.68	0.10
**BMI (kg/m^2)**	N_{obs} (N_{miss})	21163 (2474)	20577 (2413)	586 (61)	40 (4)	546 (57)
	Mean (SD)	26.7 (5.2)	26.7 (5.2)	26.5 (5.1)	26.3 (4.9)	26.5 (5.2)
	P value			0.40	0.65	0.45
**BMI (kg/m^2), Male**	N_{obs} (N_{miss})	8705 (1038)	8458 (1013)	247 (25)	14 (1)	233 (24)
	Mean (SD)	26.9 (4.5)	26.9 (4.5)	26.7 (4.5)	25.7 (4.1)	26.8 (4.5)
	P value			0.52	0.30	0.68
**BMI (kg/m^2), Female**	N_{obs} (N_{miss})	12458 (1436)	12119 (1400)	339 (36)	26 (3)	313 (33)
	Mean (SD)	26.5 (5.7)	26.5 (5.7)	26.4 (5.5)	26.7 (5.3)	26.3 (5.6)
	P value			0.55	0.90	0.51
**Body Fat (%)**	N_{obs} (N_{miss})	20712 (2925)	20139 (2851)	573 (74)	40 (4)	533 (70)
	Mean (SD)	30.0 (9.6)	30.0 (9.6)	29.7 (9.5)	30.7 (10.1)	29.7 (9.5)
	P value			0.52	0.66	0.43
**Body Fat (%), Male**	N_{obs} (N_{miss})	8571 (1172)	8326 (1145)	245 (27)	14 (1)	231 (26)
	Mean (SD)	22.9 (7.0)	22.9 (7.0)	23.0 (7.0)	21.4 (6.4)	23.1 (7.0)
	P value			0.76	0.44	0.62

**Body Fat (%), Female**	N_{obs} (N_{miss})	12141 (1753)	11813 (1706)	328 (47)	26 (3)	302 (44)
	Mean (SD)	35.0 (7.9)	35.0 (7.9)	34.8 (8.0)	35.6 (8.1)	34.7 (8.0)
	P value			0.56	0.69	0.47
**Waist (cm)**	N_{obs} (N_{miss})	20966 (2671)	20387 (2603)	579 (68)	39 (5)	540 (63)
	Mean (SD)	89.1 (14.1)	89.1 (14.1)	88.4 (14.0)	89.2 (14.9)	88.4 (13.9)
	P value			0.25	0.97	0.23
**Waist (cm), Male**	N_{obs} (N_{miss})	8625 (1118)	8381 (1090)	244 (28)	14 (1)	230 (27)
	Mean (SD)	94.6 (12.4)	94.6 (12.4)	94.3 (11.4)	93.0 (10.1)	94.4 (11.4)
	P value			0.72	0.62	0.80
**Waist (cm), Female**	N_{obs} (N_{miss})	12341 (1553)	12006 (1513)	335 (40)	25 (4)	310 (36)
	Mean (SD)	85.3 (13.9)	85.3 (13.9)	84.2 (14.2)	87.1 (16.8)	83.9 (13.9)
	P value			0.14	0.52	0.086
**SIMD Quintile**	N_{obs} (N_{miss})	20827 (2810)	20243 (2747)	584 (63)	38 (6)	546 (57)
5 (Least deprived)	N (%)	6491 (31.2%)	6311 (31.2%)	180 (30.8%)	7 (18.4%)	173 (31.7%)
4	N (%)	5348 (25.7%)	5210 (25.7%)	138 (23.6%)	11 (28.9%)	127 (23.3%)
2	N (%)	2937 (14.1%)	2851 (14.1%)	86 (14.7%)	4 (10.5%)	82 (15.0%)
3	N (%)	3371 (16.2%)	3267 (16.1%)	104 (17.8%)	10 (26.3%)	94 (17.2%)
1 (Most deprived)	N (%)	2680 (12.9%)	2604 (12.9%)	76 (13.0%)	6 (15.8%)	70 (12.8%)
	P value			0.71	0.26	0.73
**Smoking status**	N_{obs} (N_{miss})	22760 (877)	22137 (853)	623 (24)	40 (4)	583 (20)
non-smoker	N (%)	12020 (52.8%)	11695 (52.8%)	325 (52.2%)	16 (40.0%)	309 (53.0%)
ex-smoker	N (%)	6825 (30.0%)	6637 (30.0%)	188 (30.2%)	16 (40.0%)	172 (29.5%)
smoker	N (%)	3915 (17.2%)	3805 (17.2%)	110 (17.7%)	8 (20.0%)	102 (17.5%)
	P value			0.91	0.21	0.97

Avg., average; BP, blood pressure; N, number; obs, observations; mis, missing; SD, standard deviation; BMI, body mass index; SIMD, Scottish Index of Multiple Deprivation. Statistically significant associations at the 5% level are shown in red/bold.

S7 Table: Association between iciHHV-6 and self-reported disease in GS:SFHS

Disease	Total obs	Total with disease	iciHHV-6– N (%)	iciHHV-6+ N (%)	iciHHV-6+ OR (95% CI)	iciHHV-6A+ N (%)	iciHHV-6A+ OR (95% CI)	iciHHV-6B+ N (%)	iciHHV-6B+ OR (95% CI)
**Myocardial infarction**	22365	955 (4.3%)	930 (4.3%)	25 (4.1%)	0.98 (0.64, 1.5)	2 (5.0%)	1.11 (0.25, 4.96)	23 (4.1%)	0.97 (0.63, 1.51)
**Heart disease**	23128	881 (3.8%)	857 (3.8%)	24 (3.8%)	0.99 (0.62, 1.57)	0 (0.0%)	NA	24 (4.1%)	1.08 (0.68, 1.71)
**High blood pressure**	23128	3145 (13.6%)	3051 (13.6%)	94 (14.9%)	1.11 (0.84, 1.45)	5 (11.9%)	0.75 (0.25, 2.31)	89 (15.1%)	1.14 (0.86, 1.5)
**Stroke**	23128	313 (1.4%)	305 (1.4%)	8 (1.3%)	0.92 (0.44, 1.93)	1 (2.4%)	1.81 (0.21, 15.36)	7 (1.2%)	0.86 0.39, 1.89)
**Asthma**	23128	2582 (11.2%)	2515 (11.2%)	67 (10.6%)	0.93 (0.71, 1.24)	9 (21.4%)	2.08 (0.89, 4.90)	58 (9.9%)	0.86 (0.64, 1.16)
**COPD**	23120	243 (1.1%)	237 (1.1%)	6 (1.0%)	0.87 (0.35, 2.16)	0 (0.0%)	NA	6 (1.0%)	0.94 (0.37, 2.34)
**Diabetes**	23128	751 (3.2%)	731 (3.2%)	20 (3.2%)	0.93 (0.57, 1.51)	1 (2.4%)	0.68 (0.08, 5.63)	19 (3.2%)	0.94 (0.57, 1.56)
**Osteoarthritis**	23128	1707 (7.4%)	1666 (7.4%)	41 (6.5%)	0.86 (0.59, 1.24)	2 (4.8%)	0.56 (0.11, 2.68)	39 (6.6%)	0.88 (0.6, 1.28)
**Rheumatoid arthritis**	23128	394 (1.7%)	384 (1.7%)	10 (1.6%)	0.92 (0.47, 1.8)	0 (0.0%)	NA	10 (1.7%)	0.99 (0.51, 1.95)
**Hip fracture**	23128	102 (0.4%)	101 (0.4%)	1 (0.2%)	0.35 (0.05, 2.5)	0 (0.0%)	NA	1 (0.2%)	0.37 (0.05, 2.69)
**Depression**	23128	2111 (9.1%)	2046 (9.1%)	65 (10.3%)	1.13 (0.83, 1.54)	5 (11.9%)	1.57 (0.51, 4.79)	60 (10.2%)	1.11 (0.8, 1.52)
**Parkinson's disease**	23128	30 (0.1%)	30 (0.1%)	0 (0.0%)	NA	0 (0.0%)	NA	0 (0.0%)	NA
**Bowel cancer**	23128	113 (0.5%)	112 (0.5%)	1 (0.2%)	0.29 (0.04, 2.16)	1 (2.4%)	4.98 (0.48, 51.60)	0 (0.0%)	NA
**Breast cancer – all**	23128	305 (1.3%)	291 (1.3%)	14 (2.2%)	**1.88 (1.01, 3.52)**	1 (2.4%)	1.86 (0.12, 27.90)	13 (2.2%)	1.88 (0.98, 3.61)
**Breast cancer – female**	13625	304 (2.2%)	291 (2.2%)	13 (3.5%)	1.72 (0.9, 3.29)	1 (3.6%)	1.86 (0.18, 19.20)	12 (3.5%)	1.71 (0.87, 3.36)
**Lung cancer**	23128	38 (0.2%)	37 (0.2%)	1 (0.2%)	0.97 (0.13, 7.07)	0 (0.0%)	NA	1 (0.2%)	1.04 (0.14, 7.6)
**Prostate cancer**	23128	78 (0.3%)	75 (0.3%)	3 (0.5%)	1.56 (0.46, 5.31)	0 (0.0%)	NA	3 (0.5%)	1.68 (0.49, 5.78)

Obs, observations; COPD, chronic obstructive pulmonary disease; NA, analysis not possible due to limitations of low sample size. Models include adjustment for effect of age and sex.
Statistically significant associations at the 5% level are shown in red/bold.

S8 Table: Inheritance of iciHHV-6 from iciHHV-6-positive parents in GS:SFHS

Status	Children			Bin. *P* value*
	Total	iciHHV-6-positive Number (%)	iciHHV-6-negative Number (%)	
Parent iciHHV-6+	338	166 (49.11%)	172 (50.89%)	0.79
Parent iciHHV-6A+	22	10 (45.45%)	12 (54.55%)	0.83
Parent iciHHV-6B+	316	156 (49.37%)	160 (50.63%)	0.87
Father iciHHV-6+	148	88 (46.32%)	102 (53.68%)	0.57
Father iciHHV-6A+	16	4 (66.67%)	2 (33.33%)	0.45
Father iciHHV-6B+	132	84 (45.65%)	100 (54.35%)	0.34
Mother iciHHV-6+	190	78 (52.7%)	70 (47.3%)	0.35
Mother iciHHV-6A+	6	6 (37.5%)	10 (62.5%)	0.69
Mother iciHHV-6B+	184	72 (54.55%)	60 (45.45%)	0.27

To determine whether iciHHV-6 is inherited in a normal fashion, the proportion of iciHHV-6-positive children born to iciHHV-6-positive parents, and iciHHV-6-positive fathers and mothers, was compared in the GS:SFHS study. Only study participants were included in this analysis. *Bin. *P*-value represents a binomial test for whether the proportion of iciHHV-6-positive (or equivalently iciHHV-6-ve) children is different from 50%. Since all the *P* values are non-significant there is no evidence to suggest that the inheritance of iciHHV-6 is not 50/50, i.e., iciHHV-6 does appear to be inherited in a normal fashion.

S9 Table: Parity and age of first birth by iciHHV-6 status in BGS

Variable	Statistic	All Number (%)	iciHHV-6- Number (%)	iciHHV-6+ Number (%)	iciHHV-6A+ Number (%)	iciHHV-6B+ Number (%)
**Parity**	N_{obs} (N_{miss})	7903 (7)	7813 (7)	90 (0)	20 (0)	70 (0)
All	N (%)	7910 (100%)	7820 (100%)	90 (100%)	20 (100%)	70 (100%)
Never been pregnant	N (%)	1007 (12.7%)	998 (12.8%)	9 (10.0%)	3 (15.0%)	6 (8.6%)
0*	N (%)	228 (2.9%)	222 (2.8%)	6 (6.7%)	0 (0.0%)	6 (8.6%)
1	N (%)	973 (12.3%)	961 (12.3%)	12 (13.3%)	2 (10.0%)	10 (14.3%)
2	N (%)	3722 (47.1%)	3673 (47.0%)	49 (54.4%)	15 (75.0%)	34 (48.6%)
3	N (%)	1529 (19.3%)	1517 (19.4%)	12 (13.3%)	0 (0.0%)	12 (17.1%)
4	N (%)	357 (4.5%)	357 (4.6%)	0 (0.0%)	0 (0.0%)	0 (0.0%)
5	N (%)	65 (0.8%)	64 (0.8%)	1 (1.1%)	0 (0.0%)	1 (1.4%)
≥6	N (%)	22 (0.3%)	21 (0.3%)	1 (1.1%)	0 (0.0%)	1 (1.4%)
P value#				0.015	0.18	0.026
**Ever pregnant**	N_{obs} (N_{miss})	7903 (7)	7813 (7)	90 (0)	20 (0)	70 (0)
Never been pregnant	N (%)	1007 (12.7%)	998 (12.8%)	9 (10.0%)	3 (15.0%)	6 (8.6%)
Ever been pregnant	N (%)	6896 (87.3%)	6815 (87.2%)	81 (90.0%)	17 (85.0%)	64 (91.4%)
P value#				0.53	0.74	0.37
**Ever parous**	N_{obs} (N_{miss})	7903 (7)	7813 (7)	90 (0)	20 (0)	70 (0)
Never been pregnant or had pregnancy ≥26 weeks (nulliparous)	N (%)	1235 (15.6%)	1220 (15.6%)	15 (16.7%)	3 (15.0%)	12 (17.1%)
Pregnancy ≥26 weeks (parous)	N (%)	6668 (84.4%)	6593 (84.4%)	75 (83.3%)	17 (85.0%)	58 (82.9%)
P value#				0.77	1.00	0.74
**Parity levels combined (I)**	N_{obs} (N_{miss})	7903 (7)	7813 (7)	90 (0)	20 (0)	70 (0)
Never - 0	N (%)	1235 (15.6%)	1220 (15.6%)	15 (16.7%)	3 (15.0%)	12 (17.1%)
1 - 2	N (%)	4695 (59.4%)	4634 (59.3%)	61 (67.8%)	17 (85.0%)	44 (62.9%)
≥3	N (%)	1973 (25.0%)	1959 (25.1%)	14 (15.6%)	0 (0.0%)	14 (20.0%)
P value#				0.11	0.0060	0.61
**Parity levels combined (II)**	N_{obs} (N_{miss})	7903 (7)	7813 (7)	90 (0)	20 (0)	70 (0)
Never - 0	N (%)	1235 (15.6%)	1220 (15.6%)	15 (16.7%)	3 (15.0%)	12 (17.1%)
1-3	N (%)	6224 (78.8%)	6151 (78.7%)	73 (81.1%)	17 (85.0%)	56 (80.0%)
≥4	N (%)	444 (5.6%)	442 (5.7%)	2 (2.2%)	0 (0.0%)	2 (2.9%)
P value#				0.42	0.83	0.63
**Age at first birth (years)**	N_{obs} (N_{miss})	7881 (29)	7791 (29)	90 (0)	20 (0)	70 (0)
Nulliparous		1235 (15.7%)	1220 (15.7%)	15 (16.7%)	3 (15.0%)	12 (17.1%)
<20		367 (4.7%)	365 (4.7%)	2 (2.2%)	0 (0.0%)	2 (2.9%)
20-24		2108 (26.7%)	2089 (26.8%)	19 (21.1%)	3 (15.0%)	16 (22.9%)

25-29		2686 (34.1%)	2655 (34.1%)	31 (34.4%)	10 (50.0%)	21 (30.0%)
30-34		1119 (14.2%)	1101 (14.1%)	18 (20.0%)	3 (15.0%)	15 (21.4%)
35-39		319 (4.0%)	314 (4.0%)	5 (5.6%)	1 (5.0%)	4 (5.7%)
40+		47 (0.6%)	47 (0.6%)	0 (0.0%)	0 (0.0%)	0 (0.0%)
Missing		29 (0.4%)	29 (0.4%)	0 (0%)	0 (0%)	0 (0%)
P value[#]				0.53	0.68	0.60

*0 indicates a non-parous pregnancy, defined as <26 weeks gestation; parous refers to a pregnancy of ≥26 weeks gestation. [#]*P* values for comparisons between iciHHV-6-positive, iciHHV-6A-positive and iciHHV-6B-positive participants against iciHHV-6-negative participants using a Fisher test (categorical variables) or ANOVA (continuous variables).

S10 Table: Frequency of iciHHV-6-associated haplotypes in cases and controls in GS:SFHS

Haplotype	Distance from end of reference sequence[#]	iciHHV-6-negative Total = 19406 N (%)	iciHHV-positive Total = 36 N (%)	Chi-squared	*p* value
**A2_17p_hap_long**	296442	0 (0%)	5 (13.89%)		
**A2_17p_hap_short**	38430	217 (1.12%)	10 (27.7 %)	198.8	3.82E-45
**A4_18q_hap**	524075	81 (0.42%)	4 (11.11%)	71.43	2.87E-17
**A3_19q_hap1_variant1[§]**	230790	298 (1.54%)	6 (16.67%)	44.07	3.17E-11
**A3_19q_hap1. variant2[§]**	230790	251 (1.29%)	4 (11.11%)	19.71	9.01E-06
**A3_19q_hap1_all**	230790	549 (2.83%)	10 (27.78%)	71.41	71.41E-17

Haplotype	Distance from end of reference sequence[#]	iciHHV-6-negative Total = 19406 N (%)[¤]	iciHHV-6B-positive Total = 525 N (%)[¤]	Chi-squared	*p* value
**B6_7p_hap1.1***	565202	0 (0%)	9 (1.71%)		∞
**B6_7p_hap1.2***	1063104	0 (0%)	7 (1.33%)		∞
**B6_7p_ combined**		0 (0%)	16 (2.86%)		∞
**B6_9q_hap1_long**	1700684	3(0.02%)	72 (13.71%)	2522	<1E-200
**B6_9q_hap1_intermediate**	1083576	4 (0.02%)	127 (24.19%)	4536	<1E-200
**B6_9q_hap1_short**	442365	357 (1.84%)	193 (36.76%)	2310	<1E-200
**B6_9q_hap2_long**	1700684	0 (0%)	14 (2.67%)		∞
**B6_9q_hap2_short**	442365	179 (0.92%)	58 (11.05%)	437	4.87E-97
**B6_9q_combined**		536 (0.00047%)	251 (48%)		
**B4_11p_long**	454743	0 (0%)	14 (2.67%)		∞
**B4_11p_short**	205153	133 (0.69%)	34 (6.48%)	199.4	2.82E-45
**B8_17p_hap1.1[$]**	236099	0 (0%)	18 (3.43%)		∞
**B8_17p_hap1.2[$]**	62177	29 (0.15%)	15 (2.86%)	158.1	2.94E-36
**B8_17p_combined**		34 (0.18%)	33 (6.29%)	551.6	5.65E-122
**B5_19q_long**	1657017	0 (0%)	24 (4.57%)		∞
**B5_19q_short**	128527	232 (1.2%)	55 (10.48%)	303.7	5.15E-68
**B6_21q**	2242502	0 (0%)	10 (1.90%)		∞

[#]Positions are based on build GRCh37 of the human reference assembly. [¤]Numbers and percentages are cumulative, i.e., the number of individuals with a short haplotype includes the number with a long or intermediate haplotype. [§]Two versions of A3_19q_hap1 are described, which differ at 4 of the 8955 positions in the haplotype; removal of these four SNPs from the haplotype led to a reduction in specificity. *B6_7p_hap1.1 and B6_7p_hap1.2 share a common telomeric end but diverge at position 565202; the minor allele at this SNP identifies the 7p_hap1.1 haplotype, but a longer haplotype is required to identify the 7p_hap1.2 allele, which does not have the minor allele at 565202. [$]17p_hap1.1 and 17p_hap1.2 share a common telomeric end

but diverge after position 36567. 17p_hap1.2 has the minor allele at position 62177 and 17p_hap1.1 has the minor allele at position 236099. In total, 399 iciHHV-6B-associated haplotypes were identified in 397 of the 525 iciHHV-6B-positive individuals with host genotyping data.

More than one haplotype was identified in 13 individuals; two of these individuals had evidence of two integrated viruses. In the remaining 11, the collective data favoured one of the haplotypes/ancestral lineages. In a further three individuals, the identified haplotype was not consistent with the other available data. Haplotypes that were considered 'non-specific' were always the shorter versions. Two iciHHV-6B-associated haplotypes were identified in 11 of the iciHHV-6-negative individuals. Overall, 385 haplotypes in 383 individuals were predicted to be in LD with the integrated iciHHV-6B genome.

Although we have no formal proof that the identified haplotypes are in linkage with the integrated viral genomes, the evidence suggests that this is indeed the case. First, the haplotypes are strongly associated with HHV-6B positivity and in some instances exclusive to these individuals. Secondly, they are mutually exclusive (Figure S2). In almost all iciHHV-6A-positive individuals, we detected only one of the described haplotypes. This can be visualised on the Manhattan plots of the GWAS of iciHHV-6A-positive individuals grouped by haplotype where each subgroup is associated with only one of the peaks on the original Manhattan plot (Figure S2). Lastly, detection of an iciHHV-6B-associated haplotype was highly predictive of the assigned lineage when other data were considered (Figure S3).
